# Nanozyme-Triggered Cascade Reactions from Cup-Shaped Nanomotors Promote Active Cellular Targeting

**DOI:** 10.34133/2022/9831012

**Published:** 2022-06-19

**Authors:** Xin Wang, Zhongju Ye, Shen Lin, Lin Wei, Lehui Xiao

**Affiliations:** ^1^State Key Laboratory of Medicinal Chemical Biology, Tianjin Key Laboratory of Biosensing and Molecular Recognition, College of Chemistry, Nankai University, Tianjin 300071, China; ^2^College of Chemistry, Zhengzhou University, Zhengzhou 450001, China; ^3^Key Laboratory of Chemical Biology & Traditional Chinese Medicine Research, Key Laboratory of Phytochemical R&D of Hunan Province, College of Chemistry and Chemical Engineering, Hunan Normal University, Changsha 410082, China

## Abstract

Self-propelled nanomotors have shown enormous potential in biomedical applications. Herein, we report on a nanozyme-powered cup-shaped nanomotor for active cellular targeting and synergistic photodynamic/thermal therapy under near-infrared (NIR) laser irradiation. The nanomotor is constructed by the asymmetric decoration of platinum nanoparticles (PtNPs) at the bottom of gold nanocups (GNCs). PtNPs with robust peroxidase- (POD-) like activity are employed not only as propelling elements for nanomotors but also as continuous O_2_ generators to promote photodynamic therapy *via* catalyzing endogenous H_2_O_2_ decomposition. Owing to the Janus structure, asymmetric propulsion force is generated to trigger the short-ranged directional diffusion, facilitating broader diffusion areas and more efficient cellular searching and uptake. This cascade strategy combines key capabilities, i.e., endogenous substrate-based self-propulsion, active cellular targeting, and enhanced dual-modal therapy, in one multifunctional nanomotor, which is crucial in advancing self-propelled nanomotors towards eventual therapeutic agents.

## 1. Introduction

Nanocarriers have recently attracted great attention from diagnostic sensing to drug delivery owing to their unique advantages, for instance, high cargo payload, prolonged systemic circulation, and enhanced permeability and retention (EPR) effect [1–4]. Recently, with the merits of operational flexibility, noninvasiveness, low toxicity, and high spatiotemporal resolution, nanocarrier-based phototherapies have become an innovative strategy to achieve satisfactory therapeutic outcomes [5–12]. However, the therapeutic efficacy may be discounted due to the intrinsic limitations of monomodal therapy, for instance, the nonselectivity and strong laser intensity of photothermal therapy (PTT), as well as the hypoxic tumor microenvironment and short half-life time and limited diffusion distance of photoactivated singlet oxygen (^1^O_2_) for photodynamic therapy (PDT) [13]. Therefore, it is highly desired to develop multifunctional nanocarriers to achieve maximized synergistic therapy, especially with *in situ* oxygen production and active delivery abilities.

On the other hand, previous explorations have indicated that passive diffusion would hinder the delivery efficiency of nanocarriers, leading to weak biofilm penetration and nonspecific accumulation in biological environments [14, 15]. Active searching and efficient targeting toward lesion location remain a formidable challenge in diagnosis and treatment. Recently, self-propelled micro/nanomotors (MNMs), which convert local or external energies into mechanical motion, have emerged as a novel methodology to drive nanocargoes toward biological targets [16–25]. Particularly, the active cellular searching and internalization capabilities of MNMs can be modulated by regulating their speed and direction [26]. Furthermore, the combination of MNMs with other diagnostic agents and therapeutic strategies would provide a robust approach to develop active and multifunctional nanocarriers for various biomedical applications, such as diagnostic imaging, targeted drug delivery, and minimally invasive surgery [27–34]. Nevertheless, given the promising biomedical applications, several concerns still need to be addressed for the nanomotors, such as the complex actuation systems, unavailable exogenous fuels in biological surrounding, or cytotoxic by-products. On this account, nanozyme is emerging as an attractive candidate for driving nanomotors thanks to the attractive features including robust catalytic activity, high stability, and ease of preparation [35, 36].

**Figure 1:**
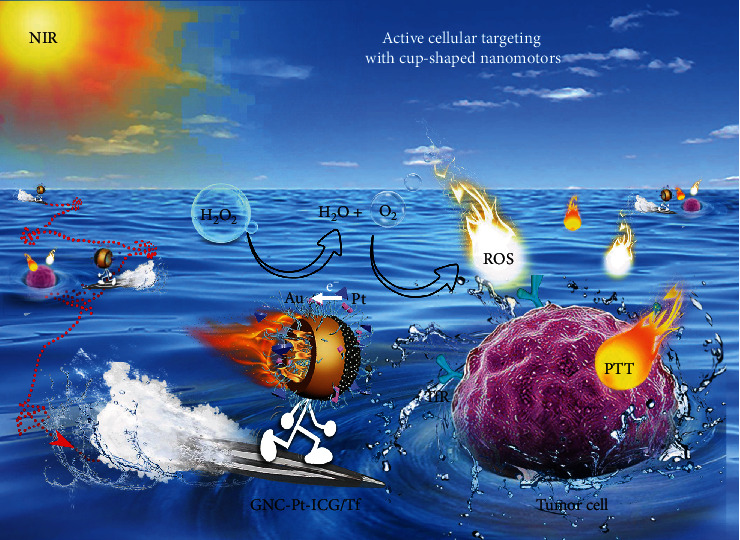
Schematic illustration of the nanozyme-powered GNCs-Pt-ICG/Tf nanomotor for enhanced dual-modal phototherapy upon NIR laser irradiation *via* a cascaded strategy consisting of the catalytic and photodynamic reactions.

Herein, we report a new design of nanomotor with good biocompatibility and robust self-propulsion capability for enhanced cell penetration, active drug delivery, and synergistic dual-modal therapy under single NIR laser irradiation. Specifically, the nanozyme-powered cup-shaped nanomotor (Figure 1) has the following features: (i) Small PtNPs *in situ* grow asymmetrically at the bottom of GNC. The Janus structure (GNC-Pt) is conducive to generate asymmetric propulsion force to break Brownian motion, resulting in short-ranged directional diffusion, which facilitates broader diffusion areas and efficient recognition toward biological targets. (ii) PtNPs with robust POD-like activity are employed as propelling elements *via* catalyzing endogenous H_2_O_2_ decomposition. Since H_2_O_2_ is overexpressed in most tumor cells [37], such endogenous H_2_O_2_-fueled nanomotor demonstrates great potential for active drug delivery in tumor environment. (iii) The GNC-Pt nanomotors serve as *in situ* O_2_ generators to improve the restriction of the hypoxia tumor microenvironment in PDT. Moreover, the active diffusion behaviors also facilitate PDT by enabling the accessibility of ICG to ^3^O_2_ and expanding the effective diffusion distance for ^1^O_2_. Taken together, we demonstrate the excellent performance of nanozyme-powered GNC-Pt nanomotors as active nanocarriers for efficient cellular uptake and enhanced synergistic PDT/PTT, providing insightful perspectives for the fabrications of active and hybrid nanomotors in a variety of biomedical applications.

## 2. Results

### 2.1. Preparation and Characterization of GNCs-Pt

**Figure 2:**
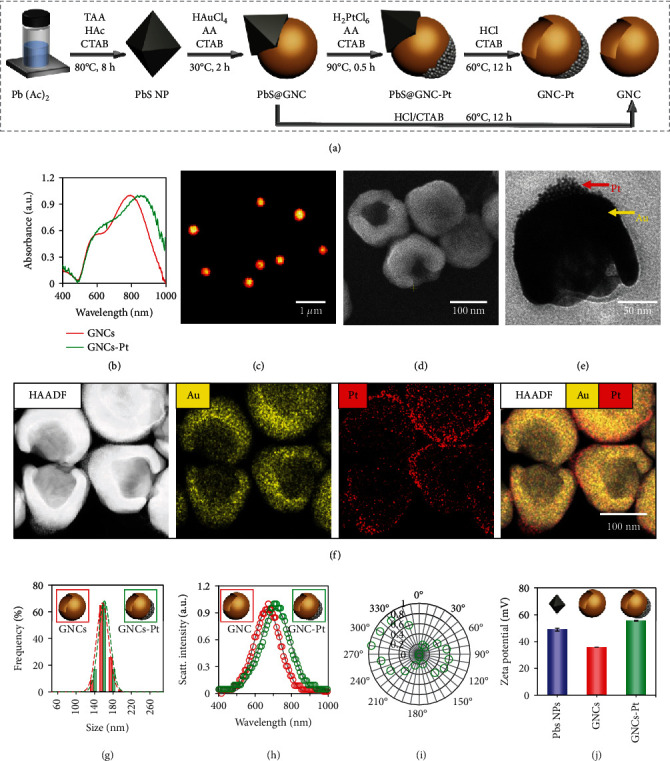
The preparation and characterization of GNCs-Pt. (a) Schematic representation of the preparation of GNCs-Pt. (b) UV-vis spectra of GNCs (red) and GNCs-Pt (green). Dark-field optical microscopic (c), SEM (d), and TEM (e) images of GNCs-Pt. (f) HAADF-STEM images and corresponding elemental maps of GNCs-Pt. (g) Size distribution of GNCs (red, 149 ± 16 nm) and GNCs-Pt (green, 154 ± 11 nm) determined by SEM images (based on 150 particles). Data are represented as mean ± SD. (h) Single-particle scattering spectra of GNCs (red) and GNCs-Pt (green). The gray line is the fitted curve based on Gaussian function. (i) The polarization-dependent scattering response (green circles) from a single GNC-Pt as a function of the angle relative to the optical axis of the polarizer. (j) Zeta potential of hexadecyl trimethyl ammonium bromide (CTAB) stabilized PbS NPs (blue, 48.9 ± 1.1 mV), GNCs (red, 35.8 ± 0.1 mV), and GNCs-Pt (green, 55.4 ± 0.3 mV). Inset: schematic diagrams of corresponding nanomaterials.

As illustrated in Figure 2(a), GNCs-Pt were fabricated by a facile bottom-up approach. Firstly, GNCs were prepared using octahedral PbS nanoparticles (PbS NPs) as the sacrificial templates (PbS@GNCs). To achieve optimal photothermal effect for PTT upon 808 nm laser irradiation, the localized surface plasmon resonance (LSPR) band of GNCs was modulated to ~800 nm by precisely adjusting the opening size (Figure S1). Subsequently, to achieve H_2_O_2_-fueled self-propulsion, small PtNPs (~2 nm) grew asymmetrically at the bottom of as-prepared PbS@GNCs (PbS@GNCs-Pt) through reducing H_2_PtCl_6_ by ascorbic acid. Finally, GNCs-Pt were obtained by selectively dissolving PbS NPs with HCl. Different from the template-assisted method, the asymmetric growth of gold and PtNPs can be precisely deposited at the high energy sites on PbS NPs and PbS@GNCs, respectively [38]. As a consequence, this method provides favorable conditions for large-scale preparation of GNC-Pt nanomotors.

The strong extended ultraviolet-visible (UV-vis) absorption band of GNC-Pt from 600 to 900 nm indicates the efficient photothermal conversion capability under NIR irradiation (Figure 2(b)). The uniform scattering color and evenly distributed scattering signal in the dark-field microscopic image confirm the excellent monodispersity of GNC-Pt (Figures 2(c) and 2(h)). Additionally, the well-defined cup-shaped structure of GNC-Pt was revealed by scanning electron microscopy (SEM) and high resolution transmission electron microscopy (TEM) (Figures 2(d) and 2(e)). Furthermore, the asymmetric decoration of PtNPs at the bottom of GNCs was confirmed by high-angle annular dark-field scanning transmission electron microscopy (HAADF-STEM) imaging and corresponding elemental mapping (Figure 2(f)). The average size of GNCs-Pt is 154 ± 11 nm based on a statistical analysis from 150 particles in the SEM images (Figure 2(g)). The orientation-dependent dipole patterns in polarization modulation experiments also verify the asymmetric structure of GNCs-Pt (Figures 2(i) and S2) [39]. Meanwhile, the zeta potential analysis (55.4 ± 0.3 mV) suggests the good stability of GNCs-Pt in water (Figure 2(j)). Similarly, detailed characterizations of PbS NPs and GNCs were also carried out to confirm the preparation processes of GNCs-Pt (Figures S2 and S3).

### 2.2. POD-Like Activity of GNCs-Pt

**Figure 3:**
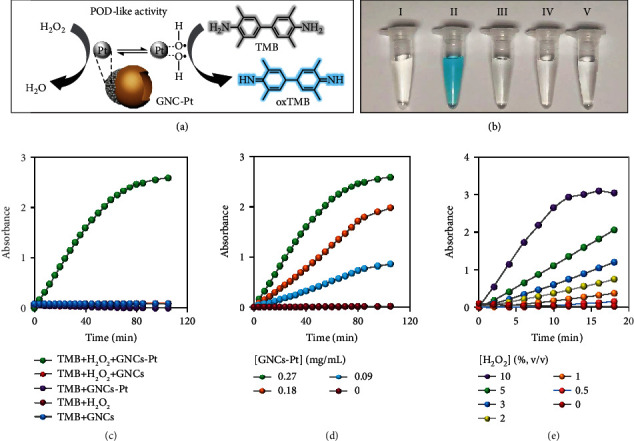
The POD-like activity of GNCs-Pt. (a) Schematic illustration of the POD-like activity of GNCs-Pt with TMB as the substrate. (b) Optical images of the oxTMB produced under different catalytic conditions for 30 min. (I) TMB + H_2_O_2_, (II) GNCs - Pt + H_2_O_2_ + TMB, (III) GNCs - Pt +TMB, (IV) GNCs + H_2_O_2_ + TMB, (V) GNCs + TMB. (c) The TMB oxidation reactions in GNCs or GNCs-Pt solutions with and without H_2_O_2_ (1%) in the disodium hydrogen phosphate-citric acid buffer (0.1 M, pH 3.0). [GNCs‐Pt] = 0.27 mg/mL, [GNCs] = 0.27 mg/mL, [TMB] = 1.0 mM. (d) and (e) Effects of the concentrations of GNCs-Pt and H_2_O_2_ on the POD-like activity.

It has been reported that H_2_O_2_ is overexpressed and accumulated during the carcinogenesis of normal cells, which can be used to fuel the nanomotors [40]. The POD-like activity of GNCs-Pt was examined with 3,5,3′,5′-tetramethylbenzidine (TMB) as the substrate (Figure 3(a)). As shown in Figure 3(b), only GNCs-Pt could efficiently catalyze the oxidation of TMB (oxidized TMB, oxTMB) in the presence of H_2_O_2_ (1%, *v*/*v*). Negligible oxTMB was observed for TMB treated with GNCs-Pt, GNCs+H_2_O_2_, GNCs, and H_2_O_2_, respectively. Additionally, the time-dependent absorbance changes of oxTMB at 652 nm in the above samples are in good agreement with the optical images (Figure 3(c)). The catalytic rate is dependent on the concentrations of GNCs-Pt and H_2_O_2_ (Figures 3(d) and 3(e)). The steady-state kinetics was also investigated to demonstrate the excellent catalytic activity of GNCs-Pt quantitatively (Figure S4 and Table S1). In addition, GNCs-Pt exhibit high catalytic activity in a broad pH range (pH = 3 ~ 9) (Figure S5). This merit overcomes the pH limitation of natural enzyme-based nanomotors for biological applications in the acidic tumor microenvironment. These results demonstrate that PtNPs endow GNCs-Pt with excellent POD-like activity, providing an essential prerequisite for self-propulsion by consuming the overexpressed endogenous H_2_O_2_ in tumor microenvironments.

### 2.3. Active Movement of GNCs-Pt

**Figure 4:**
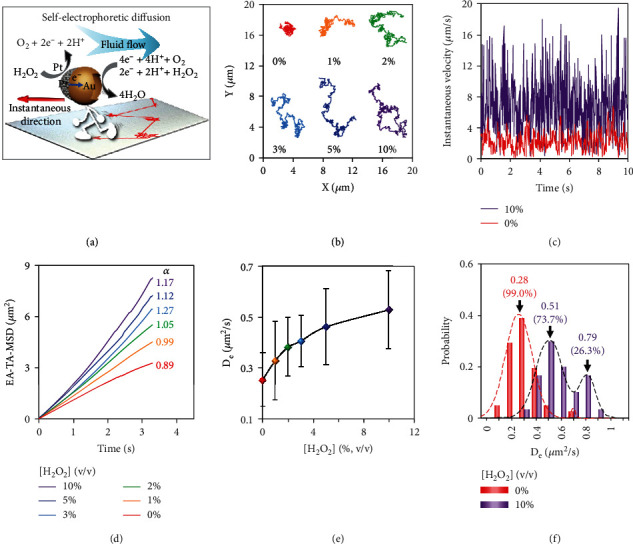
Enhanced diffusion of GNCs-Pt in long term with different H_2_O_2_ concentrations (0, 1, 2, 3, 5, and 10%). (a) Illustration of the self-electrophoresis of a GNC-Pt *via* catalyzing the decomposition of H_2_O_2_. A gradient of electric charge density will be generated across the GNC-Pt as reaction proceeds. Electroosmotic flow induced by the charge imbalance will then drive GNCs-Pt to move in the direction opposite to that of the fluid flow (red arrow). (b) Trajectories (for 10 s), (c) instantaneous velocity, and (d) EA-TA-MSD of GNCs-Pt at different H_2_O_2_ concentrations. (e) Dependence of D_e_ of GNCs-Pt with different H_2_O_2_ concentrations. (f) The distributions of *D*_*e*_ of GNCs-Pt with different H_2_O_2_ concentrations. The dashed lines are the fitted curve based on Gaussian function.

The active motion of nanomotors has proven to promote cell targeting in biological environments [26, 41]. Disclosing the effect of H_2_O_2_ on the self-propulsion capability of GNCs-Pt becomes significant and imperative. On this basis, the diffusion behaviors of GNCs-Pt at different H_2_O_2_ concentrations (0, 1, 2, 3, 5, and 10%) were investigated by single-particle tracking (SPT) (Figure 4(a)). As shown in Figure 4(b), representative trajectories of individual GNCs-Pt in a set of H_2_O_2_ solutions with different concentrations were recorded by an upright dark-field optical microscope [42]. With the concentration of H_2_O_2_ increased from 0 to 10%, the average diffusion area of GNCs-Pt greatly expands more than 25 folds from 2.41 to 61.38 *μ*m^2^ during 10 s, and the averaged velocity also increases simultaneously (Figures S6 and S7). In particular, the instantaneous velocity accelerates nearly ten folds (up to 19.5 *μ*m/s, a speed of 127 body lengths per second) due to the robust POD-like activity of GNCs-Pt (Figure 4(c)). Additionally, the ensemble-time-averaged mean-squared displacement (EA-TA-MSD) and the corresponding effective diffusion coefficient (*D*_*e*_) and anomalous exponent (*α*) of GNCs-Pt were calculated (Table S2) [43]. Basically, the diffusion modes can be categorized by *α*: subdiffusion (*α* < 1), Brownian motion (*α* ≈ 1), and superdiffusion (*α* > 1) [44, 45]. As depicted in Figure 4(d), the curves of EA-TA-MSD versus time interval transform from linear (*α* ≈ 1) to parabolic shape (*α* = 1.17) as the concentration of H_2_O_2_ increasing from 0 to 10%, indicating the transition from random Brownian motion to certain directional superdiffusion due to the enhanced self-propulsion. Meanwhile, *D*_*e*_ increases rapidly and then reaches a plateau (0.51 *μ*m^2^/s) with a gradually expanded distribution (Figures 4(e) and S8). Interestingly, a second peak of *D*_*e*_ appears at 0.79 *μ*m^2^/s in 10% H_2_O_2_ solution, suggesting the enhanced heterogeneity of self-propulsion (Figure 4(f)). In sharp contrast, there is no discernable differences in *α* and *D*_*e*_ for GNCs with or without H_2_O_2_ (10%) because of the negligible POD-like activity of GNCs (Figure S9). Therefore, GNCs-Pt exhibit H_2_O_2_-dependent enhanced motility, resulting in expanded diffusion area for target searching.

**Figure 5:**
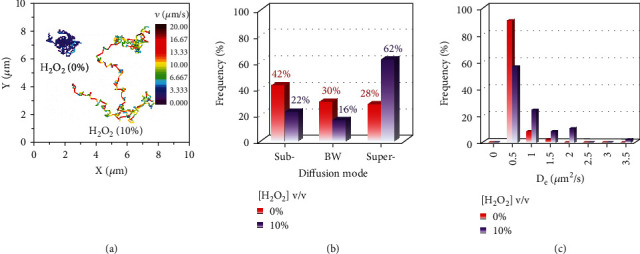
The temporal heterogeneity of diffusion behaviors of GNCs-Pt in the absence or presence of H_2_O_2_ (10%). (a) Trajectories of GNCs-Pt with the color-coded speed during 10 s. The color bar from purple to deep red represents the speed from 0 to 20 *μ*m/s. (b) The distributions of diffusion modes including subdiffusion, Brownian motion (BW), and superdiffusion. (c) The distributions of *D*_*e*_ of GNCs-Pt by a moving time-window method (1 s).

Interestingly, it is noteworthy to mention that EA-TA-MSD exhibits some disparate behaviors from the ensemble-averaged MSD (EA-MSD), indicating the time-dependent heterogeneous of the self-propulsion behaviors within a single trajectory (Figure S10) [46]. The trajectories with or without H_2_O_2_ (10%) are illustrated *via* color-coded speed (Figure 5(a)). The nanomotor moves in a manner similar to waiting-hopping as it was confined by the crowded medium, which is essential for efficient searching (more examples are shown in Figure S11). Although the SPT technique has been used to reveal the heterogeneous behaviors between individuals, the precise characteristics and dynamics of individual nanoparticle at different stages are still ignored, such as diffusion mode alternation. This can be concealed by ensemble-averaged measurement over a long period of time. To address this limitation and reveal the directionality of the nanomotors, we further investigated individual trajectories by a moving time-window method.

A typical trajectory of GNCs-Pt in 10% H_2_O_2_ was divided into 10 pieces sequentially by a moving time-window of 1.0 s, and time-averaged MSD (TA-MSD) was also calculated in each window (Figures S12-14). As shown in Figure 5(b), the statistical results according to a series of trajectories at different H_2_O_2_ concentrations were obtained. It turns out that although the dominant diffusion mode is H_2_O_2_-dependent, GNCs-Pt normally undergo three diffusion modes alternately rather than one or two of them. The higher H_2_O_2_ concentration, the greater probability of superdiffusion with higher D_*e*_ is observed, providing promising potential for active transport in tumor environment by utilizing overexpressed endogenous H_2_O_2_ (Figure 5(c)).

The searching efficiency of nanoparticles is determined by their diffusion behaviors, such as Brownian motion, Lévy walk, and Lévy flights [47]. To further understand the influence of H_2_O_2_ on the directionality in the diffusion process, the distribution of azimuthal angle displacement (*φ*) of GNCs-Pt in solution with different H_2_O_2_ concentrations was examined [48]. For comparison, we took the same trajectories in Figure S12 as examples. Interestingly, GNCs-Pt undergo more directional diffusion during each time window in 10% H_2_O_2_, which is averaged in the whole trajectory analysis (Figure S15). In sharp contrast, the isotropic random Brownian motion of GNCs-Pt without H_2_O_2_ is observed *via* the moving-window analysis or whole trajectory analysis (Figure S16). Taken together, these results illustrate that GNCs-Pt possess H_2_O_2_-dependent accelerated and short-ranged directional diffusion, which can greatly expand the searching area and facilitate cellular recognition and membrane penetration performance [49]. Meanwhile, the generated O_2_ can modulate the hypoxia tumor microenvironment, which holds great potential for enhanced PDT.

### 2.4. Tf and ICG Loading and Characterization

Inspired by the enhanced self-propulsion movability and O_2_ production ability, we conceive that GNCs-Pt can serve as active nanocarriers for synergistic PDT/PTT under NIR laser irradiation. Briefly, ICG with excitation wavelength at ~800 nm was loaded on GNCs-Pt (GNCs-Pt-ICG) *via* electrostatic adsorption for efficient photodynamic reaction by taking full use of the produced O_2_. Although the enhanced self-propelled movability could increase the searching efficiency and drive the nanomotor toward biological targets, it is still difficult to bind on the cell membrane and be internalized by cancer cells due to the lack of specific recognition and binding capability. Because Tf receptor (TfR) is overexpressed on most of cancer cell membrane, we decorate Tf on the nanomotors (GNCs-Pt-ICG/Tf) to improve the recognition ability toward cancer cells. Furthermore, the nanomotors were modified with methoxy polyethylene glycol thiol (mPEG-SH) to improve the colloidal stability and reduce the cytotoxicity in biological applications. As a control, GNCs were also modified with the same methods (GNCs-ICG/Tf). The successful decorations of ICG and Tf have been proved by UV-vis absorption spectra, zeta potential analysis, and Fourier transform infrared (FT-IR) spectroscopy (Figure S17, Table S3). In addition, the loading capacity is 25.97 mg ICG (33.51 *μ*mol) for 1.0 g GNCs-Pt (Figure S18).

### 2.5. ^1^O_2_ Generation

**Figure 6:**
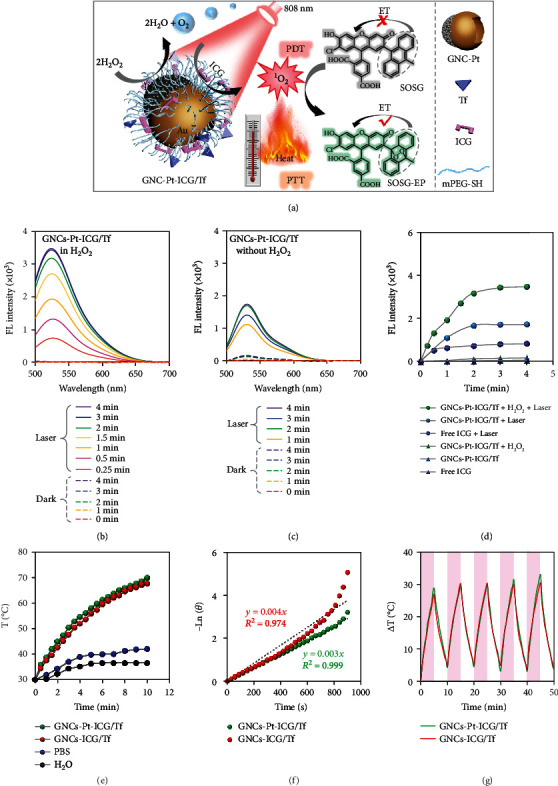
The ROS generation and photothermal properties of GNCs-Pt-ICG/Tf. (a) Schematic illustration of the mechanism of GNCs-Pt-ICG/Tf for synergistic PDT/PTT upon NIR laser irradiation *via* a cascade reaction. (b)–(d) The ROS generation ability of GNCs-Pt-ICG/Tf with SOSG as an indicator. (b) GNCs-Pt-ICG/Tf in the presence of H_2_O_2_ (1%) with and without 808 nm laser. (c) GNCs-Pt-ICG/Tf in the absence of H_2_O_2_ with and without 808 nm laser. (d) GNCs-Pt-ICG/Tf and free ICG in different conditions. [nanomaterials] = 270 *μ*g/mL; [ICG] = 9.05 *μ*M; laser: 808 nm, 2 W/cm^2^. (e)–(g) Photothermal properties of GNCs-Pt-ICG/Tf and GNCs-ICG/Tf. (e) Temperature evaluation of GNCs-Pt-ICG/Tf, GNCs-ICG/Tf, PBS, and deionized water with 808 nm laser irradiation for different times. (f) A plot of −*lnθ* versus time obtained from the cooling period for 15 min. (g) The photostability of GNCs-Pt-ICG/Tf and GNCs-ICG/Tf in PBS with 808 nm laser on and off for five cycles. [nanomaterials] = 137 *μ*g/mL; PBS: 10 mM, pH = 7.4; laser: 808 nm, 2 W/cm^2^.

Abnormal metabolism of cancer cells leads to the accumulation of H_2_O_2_ in tumor environment. Because of the POD-like activity, GNCs-Pt-ICG/Tf can serve as O_2_ generators to promote the PDT effect by consuming overexpressed H_2_O_2_.  Figure 6(a) illustrates the cascade concept of the catalytic decomposition of H_2_O_2_ and the enhanced photodynamic reaction of ICG. To verify this conceive, ^1^O_2_ production ability of GNCs-Pt-ICG/Tf was studied under 808 nm laser irradiation (2 W/cm^2^) by using singlet oxygen sensor green (SOSG) as the indicator (Figure 6(b)). The slight ^1^O_2_ generation by GNCs-Pt-ICG/Tf without H_2_O_2_ can be attributed to the photodynamic reaction of the loaded ICG with residual O_2_ in PBS (Figure 6(c)). However, as a control, ^1^O_2_ generated from equivalent free ICG (9.05 *μ*M) was much lower than that of GNCs-Pt-ICG/Tf with the same laser irradiation, which can be ascribed to the intrinsic poor solubility and stability of free ICG (Figures 6(d) and S19). As shown in Figure 6(d), only in the presence of both laser (808 nm, 2 W/cm^2^) and H_2_O_2_ (1%), the fluorescence intensity of SOSG sharply increased over 2 times than that of GNCs-ICG/Tf, indicating the generated O_2_ from the first stage of cascade reaction could accelerate ^1^O_2_ generation. This holds promising potentials in enhanced PDT due to the following three points: (1) the consecutive generation of O_2_ addresses the inherent limitation of hypoxia tumor environment; (2) the challenge of poor solubility and biological stability of ICG is greatly improved by the nanomotor; and (3) the active diffusion behaviors enable the accessibility of ICG to ^3^O_2_ and expand the effective diffusion distance for ^1^O_2_.

### 2.6. Photothermal Performance

Because of the strong absorption in the NIR region, GNCs-Pt-ICG/Tf would possess good photothermal conversion efficiency for potential tumor treatment. As shown in Figure 6(e), GNCs-Pt-ICG/Tf and GNCs-ICG/Tf with concentration of 137 *μ*g/mL were irradiated with 808 nm laser (2 W/cm^2^) for 10 min. The temperature of GNCs-Pt-ICG/Tf and GNCs-ICG/Tf solution increased from 30°C to 74.4°C and 73.3°C, respectively. However, under the same conditions, the temperature of phosphate buffer saline (PBS) solution and deionized water only ascended to 42.0°C and 36.5°C, respectively. The photothermal conversion efficiencies of GNCs-Pt-ICG/Tf and GNCs-ICG/Tf are calculated to be 44.31% and 41.09%, respectively, which are comparable to that of commonly used nanomaterials for PTT such as gold nanorods (39.2%) [50], Cu_3_BiS_3_ nanorods (40.7%) [51], and Pt-CuS nanoparticles (34.5%) (Figures 6(f) and S20) [52]. These results indicate that the deposition of PtNPs has negligible influence on the photothermal performance of GNCs. In addition, there was negligible temperature deterioration in these two samples during the five “on/off” irradiation cycles, indicating the excellent photothermal stability and reproducibility of GNCs (Figure 6(g)). All these results demonstrate that GNCs-Pt-ICG/Tf would be a promising candidate for photothermal applications.

### 2.7. Biological Stability and Cytotoxicity

**Figure 7:**
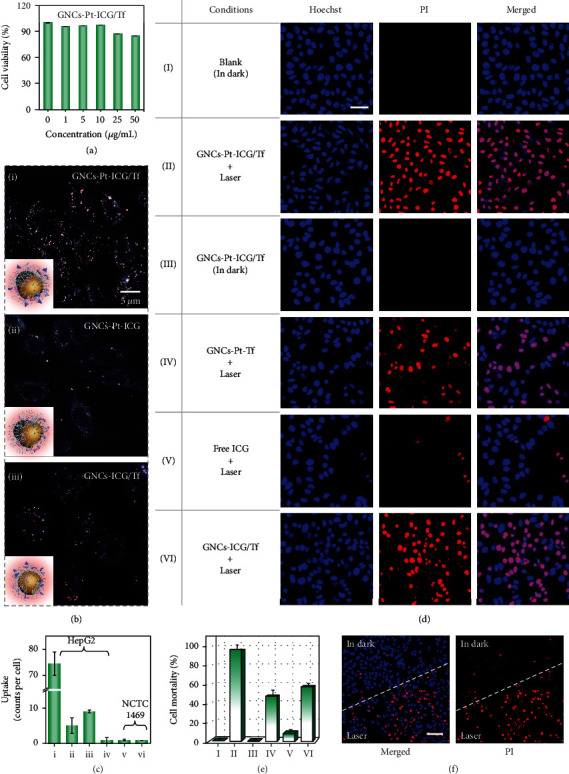
Cytotoxicity and antitumor efficacy of GNCs-Pt-ICG/Tf *in vitro*. (a) HepG2 cell viability incubated with GNCs-Pt-ICG/Tf of various concentrations (0, 1, 5, 10, 25, and 50 *μ*g/mL) for 24 h. Error bars represent the mean ± SD (*n* = 5). (b) The effects of PtNPs and Tf/ICG modification on the cellular uptake efficiency of GNCs-Pt-ICG/Tf. Inset: schematic diagrams of the corresponding nanomaterials. (c) The cellular uptake capability of (i) GNCs-Pt-ICG/Tf, (ii) GNCs-Pt-ICG, (iii) GNCs-ICG/Tf, and (iv) GNCs-ICG for HepG2 cells and (v) GNCs-Pt-ICG/Tf and (vi) GNCs-Pt-ICG for NCTC1469 cells, respectively. Confocal laser scanning microscopy (CLSM) images (d) and corresponding cell mortality (e) of HepG2 cells treated with (I) culture medium, (II) GNCs-Pt-ICG/Tf+laser, (III) GNCs-Pt-ICG/Tf (in dark), (IV) GNCs-Pt-Tf+laser, (V) free ICG+laser, and (VI) GNCs-ICG/Tf+laser, respectively. Scale bar: 50 *μ*m. (f) CLSM images of HepG2 cells cocultured with GNCs-Pt-ICG/Tf at the edge of laser irradiation. Scale bar: 100 *μ*m. [nanomaterials] = 50 *μ*g/mL; [ICG] = 1.68 *μ*M; Laser: 808 nm, 2 W/cm^2^.

Good biological stability and biocompatibility are two essential factors to evaluate the performance of nanoparticles in biological applications. The stability of GNCs-Pt-ICG/Tf was explored with dark-field optical microscopy at the single-particle level. GNCs-Pt-ICG/Tf display good monodispersity in H_2_O, PBS, and Dulbecco's Modified Eagle Medium (DMEM) (Figure S21). In contrast, obvious aggregations from GNCs-Pt (stabilized by CTAB) in PBS and DMEM were observed (Figure S22). Subsequently, the cytotoxicity of GNCs-Pt-ICG/Tf was evaluated using the standard 3-(4,5-dimethylthiazol-2-yl)-2,5-diphenyltetrazolium bromide (MTT) assay (Figure 7(a)). HepG2 cells were cultured with different concentrations of GNCs-Pt-ICG/Tf (0, 1, 5, 10, 25, and 50 *μ*g/mL) in dark for 24 h. The survival rate of HepG2 cells is higher than 85% even at a high concentration of 50 *μ*g/mL. In sharp contrast, distinct cytotoxicity (11.5% cell viability) from GNCs-Pt was observed at a low concentration of 5.0 *μ*g/mL (Figure S23). These results show that modification of mPEG-SH is necessary to improve the biostability and biocompatibility of nanomotors (Figures S24 and S25).

### 2.8. Cell Targeting and Uptake

As efficient cellular targeting and internalization are important for cancer treatment, the self-propelled diffusion of GNCs-Pt-ICG/Tf on HepG2 cell membrane before internalization was recorded and analyzed by SPT. Much broader diffusion area and faster instantaneous velocity and *D*_*e*_ from GNCs-Pt-ICG/Tf (*v*_max_ = 8.31 *μ*m/s, *D*_*e*_ = 0.031 *μ*m^2^/s) were observed than those of GNCs-ICG/Tf on living cell membrane (*v*_max_ = 2.95 *μ*m/s, *D*_*e*_ = 0.006 *μ*m^2^/s) (Figure S26). These results clearly demonstrate that the self-propulsion can noticeably increase the active diffusion of nanomotors in biological media.

In addition, cellular uptake of GNCs-Pt-ICG/Tf was investigated in HepG2 cells with dark-field microscopy (Figure 7(b)). The amount of GNCs-Pt-ICG/Tf within the cells can be counted individually under low incubation dosage. According to the single-particle counting results, the number of GNCs-Pt-ICG/Tf in HepG2 cells is higher than that of GNCs-ICG (over 80 folds) or GNCs-ICG/Tf (over 10 folds), respectively, indicating that the self-propelled diffusion of nanomotors could significantly promote the cellular recognition and uptake (Figure 7(c)). As a control, the number of internalized GNCs-Pt-ICG (without Tf) is far fewer than that of GNCs-Pt-ICG/Tf, which is because the active diffusion only enhances the accessibility to the cell membrane, but not the uptake efficacy. In other words, the functionalization of nanomotors with Tf is crucial for cellular recognition and uptake, while the accelerated movement of nanomotor could improve these processes. In addition, NCTC1469 cells (a mouse fibroblasts cell line) with negligible surface expression of TfR were selected as another control to explore the specific recognition ability of GNCs-Pt-ICG/Tf toward cancerous cells (Figure S27). As expected, the number of GNCs-Pt-ICG/Tf in the NCTC1469 cells is much less than in the HepG2 cells.

### 2.9. Enhanced Dual-Modal Phototherapy by NIR Irradiation

Encouraged by the performances of GNCs-Pt-ICG/Tf in active cellular recognition and uptake, as well as photothermal and photodynamic capability, we further investigated the synergetic PDT/PTT efficacy with 808 nm laser irradiation (Figures 7(d) and 7(e)). The therapeutic efficacy was examined on the basis of cell viability outcomes by treating HepG2 cells with GNCs-Pt-ICG/Tf+laser, GNCs-Pt-ICG/Tf (in dark), GNCs-Pt-Tf+laser, ICG+laser, and GNCs-ICG/Tf+laser, respectively. As expected, negligible toxicity was detected when the cells were incubated with GNCs-Pt-ICG/Tf without 808 nm laser irradiation, which was consistent with the results in MTT assay. In contrast, ^1^O_2_ generation capability of GNCs-Pt-ICG/Tf is activated upon 808 nm laser irradiation (2 W/cm^2^, 5 min), resulting in apoptosis for more than 96.4% of cells. Moreover, the mortality of the cells incubated with GNCs-Pt-ICG/Tf is distinctly higher than the total mortality with GNCs-Pt-Tf (47.4%) and free ICG (8.5%) groups, suggesting that the cascade strategy can greatly enhance the PDT/PTT compared to that of single therapeutic model. Furthermore, half of the cells (treated with GNCs-Pt-ICG/Tf) within an observation window were illuminated with laser, and then, the images of cells in the laser edge area were also captured (Figure 7(f)). There is a clear dividing line, indicating the negligible cytotoxicity of GNCs-Pt-ICG/Tf in dark, while the potent therapeutic effect upon 808 nm laser irradiation.

## 3. Discussion

In summary, we introduce a nanozyme-powered cup-shaped nanomotor (GNCs-Pt-ICG/Tf) *via* a facile bottom-up method for enhanced synergistic PDT/PTT upon NIR laser irradiation. The asymmetric growth of PtNPs endowed the nanomotor with accelerated (up to 19.5 *μ*m/s) and short-ranged directional self-propelled diffusion by catalyzing the decomposition of overexpressed endogenous H_2_O_2_. This feature boosts the diffusion area and recognition efficiency. As a result, the cellular uptake efficiency of GNCs-Pt-ICG/Tf by HepG2 cells is around 10 folds higher than that of GNCs-ICG/Tf. Meanwhile, the generated O_2_ promotes the photodynamic reaction of ICG, which enhances the PDT effect by overcoming the inherent limitation of hypoxia in tumor environments. Furthermore, the efficient photothermal conversion of GNCs-Pt-ICG/Tf enables the synergistic phototherapy, resulting in the distinctly higher cell mortality after treatment (96.4%). Such a cascade strategy consisting of nanozyme reaction and photodynamic reaction can be generalized to other types of nanomaterials (e.g., Au, Fe_3_O_4_, and Cu_x_O nanoparticles) or reactions (e.g., Fenton-like reaction). The efficient cellular targeting and boosted dual-modal phototherapy achieved by the nanozyme-powered nanomotor provides a new strategy of designing multifunctional nanocarriers in a controlled and active manner.

## 4. Materials and Methods

### 4.1. Materials

Unless otherwise noted, the reagents were purchased from commercial sources and used directly without further purification. Lead acetate (Pb(AC)_2_, 99.5%), acetic acid (HAc), thioacetamide (TAA), cetyltrimethylammonium bromide (CTAB), chloroauric acid (HAuCl_4_•3H_2_O, ≥99.9%), hydrochloric acid (HCl, 36%~38%), chloroplatinic acid hexahydrate (H_2_PtCl_6_•6H_2_O, ≥99.9%), ascorbic acid (AA, ≥99.0%), methoxy polyethylene glycol thiol (mPEG-SH, MW≈6000), N-hydroxysuccinimide polyethylene glycol thiol (NHS-PEG-SH, MW≈6000), and polyetherimide (PEI, MW≈6000) were purchased from Aladdin Reagent Co. Ltd (Shanghai, China).

Hydrogen peroxide (H_2_O_2_, 30 wt.% in H_2_O), indocyanine green (ICG), and human transferrin (Tf, MW≈79 kD, 98%) were bought from Sigma-Aldrich (St. Louis, Mo, USA). Bis(*p*-sulfonatophenyl)phenylphosphine dihydrate dipotassium salt (BSPP, 97%) and tri(hydroxymethyl) amino methane hydrochloride (Tris-HCl, 99%) were purchased from J&K Scientific (Beijing, China). Dulbecco's Modified Eagle Medium (DMEM), penicillin/streptomycin (PS, 100×), fetal bovine serum (FBS), and trypsin were brought from Gibco (Carlsbad, USA). Singlet oxygen sensor green (SOSG) was purchased from Invitrogen (Carlsbad, USA). Methyl thiazolyl tetrazolium, Hoechst 33342 (100×), and propidium iodide (PI) were obtained from Beyotime Biotechnology (Haimen, China). Besides, deionized (DI) water with a resistivity of 18.1 M*Ω* cm was used in all relevant experiments.

### 4.2. Instruments

Ultraviolet-visible (UV-vis) absorption spectra were recorded using a UV-2450 spectrophotometer (Shimadzu, Tokyo, Japan) in a standard quartz cuvette with 1 cm path length. Scanning electron microscopy (SEM) images were captured by an Apreo S LoVac SEM at 2 kV (FEI, Hillsboro, USA). Transmission electron microscope (TEM) images were recorded using a JEM2100 instrument (JEOL, Tokyo, Japan). High-resolution (HR) TEM images and elemental mapping were acquired *via* a Talos F200X G2 instrument (FEI, AEMC, Hillsboro, USA). Zeta potential was measured *via* a laser light scattering spectrometer (NanoBrook 173plus and ZetaPals/BI-200SM, New York, USA). Infrared spectra were performed on a Fourier transform infrared (FT-IR) spectrometer (Nicolet AVATAR-360, ThermoFisher, USA). The dark-field microscopic imaging experiments were carried out using a Nikon Eclipse Ni-U upright optical microscope (Nikon, Tokyo, Japan) with a laser beamsplitter (20 × 20 mm, Edmund Optics, Barrington, USA). The images were collected by a high-resolution color microscope camera (Digiretina 16, Xintu Optoelectronics Co., LTD, Fujian, China). The trajectories of nanomotors were captured by a sCMOS camera (Orcaflash 4.0, Hamamastu, Japan). Furthermore, the polarization-dependent scattering signals of single nanoparticle were recorded with a rotating polarizer. Confocal fluorescent images were obtained with a confocal laser scanning microscope (CLSM, A1R+, Nikon, Tokyo, Japan). Temperatures were determined using an infrared temperature sensor (XINTEST HT-20, Guangzhou, China). The optical density (OD) values of blue oxidized TMB (oxTMB) and MTT was measured on a microplate reader (Sunrise, Tecan, Austria).

### 4.3. Preparation of Nanozyme-Powered GNCs-Pt Janus Nanomotors

Firstly, Au selectively grew at one vertex of each octahedral PbS nanoparticles (PbS NPs), which was controlled by electron transfer from PbS to Au during Au nucleation (PbS@GNCs). Briefly, aqueous solutions of CTAB (0.68 mL, 0.1 M), acetic acid (HAc, 1.37 mL, 1.0 M), lead acetate (Pb(Ac)_2_, 0.68 mL, 0.5 M), thioacetamide (TAA, 0.68 mL, 0.5 M), and DI water (10.75 mL) were mixed together at 25°C. Then, the mixture was heated to 80°C for 8 h. After reaction, the obtained PbS NPs were centrifuged (5000 rpm × 10 min) to remove the excessive precursors and redispersed into DI water (15 mL) [38]. Then, H_2_PtCl_6_ (0.8 mL, 1.0 mM) was added into as-prepared PbS@GNCs solution (15 mL) and stirred at 25°C for 10 min. Then, AA (0.8 mL, 1.0 mM) was dropped slowly, and the mixture was heated to 90°C for 3 h. The PbS@GNCs-Pt were obtained by centrifugation (5000 rpm × 5 min) and redispersed in CTAB solution (15 mL, 0.1 M). Thirdly, GNCs-Pt were prepared by selectively dissolving PbS components of PbS@GNCs-Pt by HCl. Briefly, HCl (0.75 mL, 5 M) was added into the PbS@GNCs-Pt solution (15 mL). Subsequently, the obtained solution was stirred at 65°C for 12 h. The GNCs-Pt were finally obtained by centrifugation (5000 rpm × 5 min) and redispersion in DI water (15 mL).

### 4.4. Peroxidase- (POD-) like Activity

The POD-like activity of GNCs-Pt was conducted at room temperature in a 96-well plate using 3,5,3′,5′-tetramethylbenzidine (TMB, 5.0 *μ*L, 42 mM) as substrate. A series of different catalyzers (GNCs-Pt or GNCs) and concentrations of H_2_O_2_ (0, 0.5, 1, 2, 3, 5, and 10%, *v*/*v*) were added into the disodium hydrogen phosphate-citric acid buffer (0.1 M, pH 3), and the total volume of reaction systems was set to 210 *μ*L in each well. The absorption of the reaction systems was monitored at 652 nm at certain time using a microplate reader, which were further drawn into a curve to determine the POD-mimetic activity. In addition, the pH stability of GNCs-Pt was also evaluated by the above method in with pH values in the range from 1 to 11 for 30 min.

### 4.5. Self-Propulsion Diffusion Behavior Analysis

The single-particle measurements were performed on a Nikon Eclipse Ni-U upright optical microscope. Taking GNCs-Pt for example, the GNCs-Pt were firstly immobilized on the pretreated glass slide surface (22 × 22 mm^2^). Then, the scattered light from individual GNCs-Pt was measured with an objective (40×, numerical aperture (NA) = 0.75) and captured by a sCMOS camera (Orcaflash 4.0, Hamamastu, Japan. Pixel size 6.5 × 6.5 *μ*m^2^). To measure the polarization-dependent scattering signal from individual GNCs-Pt, a polarizer was put below the oil dark-field condenser. Through rotating the optical axis of the polarizer (from 0° to 360°), the orientation-dependent scattering signals from single GNCs-Pt were recorded by the sCMOS camera. All images were processed with ImageJ.

### 4.6. Motion Behaviors

Monitoring the diffusion trajectories of nanoparticles in water at the single-particle level is a great challenge because of their fast 3D Brownian motion. On this account, glycerol (50%, *v*/*v*) was added to increase the viscosity of the medium and slow down the movement of GNCs-Pt in all relevant noncell-tracking experiments. Firstly, the GNCs-Pt were mixed with a series of different concentrations of H_2_O_2_ (0, 1, 2, 3, 5, and 10%, *v*/v) and glycerol. Subsequently, the mixture was injected into the chamber. The self-propulsion diffusion of GNCs-Pt in different conditions was observed by an objective (40×, NA = 0.75). And each sample was videoed simultaneously for 10 s by a sCMOS camera (Orcaflash 4.0, Hamamastu, Japan. Pixel size 6.5 × 6.5 *μ*m^2^) with a frame rate of 49.99 fps.

### 4.7. The Fabrication of GNCs-Pt-ICG/Tf

To fabricate an active transport platform based on GNCs-Pt for synergistic enhanced photodynamic/thermal therapy, we further decorate transferrin receptor (Tf), indocyanine green (ICG), and mPEG-SH on GNCs-Pt. To decorate Tf on the surface of GNCs-Pt, Tf was functionalized with thiol group. NHS-PEG-SH (10 *μ*L, 0.5 mg/mL) and Tf (63 *μ*L, 5 mg/mL) was added into Tris HCl buffer (90 *μ*L, pH 8.5, 10 mM). The Tf-PEG-SH solution (6.0 *μ*M) was obtained after the mixture solution was shaken gently at 25°C for 2 h.

Firstly, GNCs-Pt stock solution (1 mL) was centrifuged (5000 rpm × 5 min) to remove the extra CTAB in the solution and redispersed in DI water (50 *μ*L). Subsequently, BSPP (50 *μ*L, 1 mg/mL) was added and gently stirred at 25°C for 3 h to substitute the CTAB on the surface of GNCs-Pt. The BSPP modified GNCs-Pt was obtained by centrifugation (5000 rpm × 5 min) and redispersion in DI water (100 *μ*L) [42, 44]. Then, Tf-PEG-SH solution (3 *μ*L) was gradually added and the mixture was gently stirred for additional 3 h. After that, PEI (1 *μ*L, 0.5 mg/mL) was added to endow GNCs-Pt with positive charge for ICG loading. After the addition of free ICG (7 *μ*L, 0.5 mg/mL) for 6 h, mPEG-SH (5 *μ*L, 0.5 mg/mL) was added and shaken for another 6 h to increase the stability and biocompatibility of the nanomotors. Finally, GNCs-Pt-ICG/Tf were collected by centrifugation (5000 rpm × 5 min) and suspended in DI water (100 *μ*L).

As controls, a series of nanomaterials were prepared with the same methods, such as GNCs-Pt loaded with ICG (GNCs-Pt-ICG), GNCs-Pt decorated with Tf (GNCs-Pt-Tf), GNCs loaded with Tf and ICG (GNCs-ICG/Tf), and GNCs modified with Tf (GNCs-Tf, without ICG).

### 4.8. ^1^O_2_ Generation Capability Assessment

The ^1^O_2_ production ability of GNCs-Pt-ICG/Tf was investigated with singlet oxygen sensor green (SOSG) as indicator [53]. All relevant experiments were conducted in PBS (pH 7.4, 10 mM) solution following pretreatment with nitrogen to avoid the interference from dissolved O_2_ as much as possible. A certain of GNCs-Pt-ICG/Tf (0.27 mg/mL, 9.05 *μ*M ICG loaded) was added into the mixture solution of SOSG (3 *μ*M) and H_2_O_2_ (1%, *v*/*v*). Then, the mixture was irradiated with an NIR laser (808 nm, 2 W/cm^2^). The changes in fluorescence intensity were detected at predetermined intervals with a fluorescence spectrophotometer (Ex/Em = 470/527 nm). As controls, the ^1^O_2_ production ability in free ICG (9.05 *μ*M) and GNCs-ICG/Tf (0.27 mg/mL) with different concentration of H_2_O_2_ and laser conditions was also evaluated.

### 4.9. Photothermal Performance

To examine the photothermal conversion efficiency of GNCs-Pt-ICG/Tf, GNCs-Pt-ICG/Tf solution (1 mL) was added in a quartz cuvette and exposed to a NIR laser at a power of 2 W/cm^2^ for 15 min. Then, the solution was cooled down naturally for another 15 min. The temperature changes were recorded by an infrared thermal imaging camera every 30 s. The photothermal conversion efficiency (*η*) can be calculated according to:

(1) $$\begin{array}{c} \eta =\frac{hS\;\left(T_{\max,sample}-T_{\max,H_{2}O}\right)-Q_{dis}}{I\left(1-10^{-A_{808}}\right)}, \end{array}$$

where ℎ is the heat transfer coefficient, *S* is the irradiated area, and *T*_max,*sample*_ and *T*_max,*H*_2_*O*_ are the maximum equilibrium temperature of the sample and H_2_O, respectively. *T*_*surr*_ is the ambient temperature of the surroundings (*T*_*surr*_ = 30°*C*). *Q*_*dis*_ means heat dissipation from the system to the surroundings, and it is calculated to be approximately equal to 0 mW. *I* represents the laser power (2 W/cm^2^). *A*_808_ is the sample absorbance at 808 nm.

When the heat input is equal to the heat output, *hS* is calculated with the following:

(2) $$\begin{array}{c} hS=\frac{\sum_{i}m_{i}C_{p,i}}{\tau_{s}}\approx \frac{m_{H_{2}O}∙C_{H_{2}O}}{\tau_{s}}, \end{array}$$

where *m*_*H*_2_*O*_ and *C*_*H*_2_*O*_ are the mass and thermal capacity of the water, respectively. *τ*_*s*_, the heat dissipation time constant, is calculated by plotting a linear data of cooling period with the negative natural logarithm using the following:

(3) $$\begin{array}{c} t=-\tau_{s}\ln\theta =\tau_{s}\ln\frac{T-T_{surr}}{T_{\max}-T_{surr}}, \end{array}$$

where *t* is the cooling time (*s*).

(4) $$\begin{array}{c} T_{\max,GNCs-Pt-ICG/Tf}=74.4^{{}^{\circ}}C,T_{\max,GNCs-ICG/Tf}=73.3^{{}^{\circ}}C.\\ {Abs}_{808\;nm,GNCs-Pt-ICG/Tf}=0.420,{Abs}_{808\;nm,GNCs-ICG/Tf}=0.472. \end{array}$$

Thus, according to experiments, the photothermal conversion efficiency of GNCs-Pt-ICG/Tf and GNCs-ICG/Tf under 808 nm laser (2.0 W/cm^2^) is 44.31% and 41.09%, respectively.

#### 4.9.1. Photothermal Stability

The photothermal stability of GNCs-Pt-ICG/Tf solution was measured by cycle irradiation. Briefly, the solution was irradiated with 808 nm laser at 2.0 W/cm^2^ for 10 min. Then, the laser was turned off, and the solution was cooled down to ambient temperature for another 10 min. The above procedures were repeated for 5 times, and the temperature changes were recorded by an infrared thermal imaging camera. As controls, the photothermal conversion efficiency and photothermal stability GNCs-ICG/Tf were also measured through the same methods.

### 4.10. Biological Stability

PBS (pH 7.4, 10 mM) and DMEM were used to mimic the human blood plasma environments. GNCs-Pt-ICG/Tf was first mixed with different media (H_2_O, PBS, and DMEM) for 30 min. Then, the biological stability of GNCs-Pt-ICG/Tf was investigated by a Nikon Eclipse Ni-U upright optical microscope.

#### 4.10.1. MTT Assay

The standard MTT cell assay was used to investigate the cytotoxicity of GNCs-Pt-ICG/Tf. Briefly, the HepG2 cells were first seeded in 96-well plates at a density of 4 × 10^4^ cells per well and grown in 5% CO_2_ at 37°C for 8 h. The culture medium is DMEM with FBS (10%, *v*/*v*) and PS (1%, *v*/*v*). Then, the HepG2 cells were incubated with GNCs-Pt-ICG/Tf at different concentrations (0, 1, 5, 10, 25, and 50 *μ*g/mL) for another 24 h in dark. Subsequently, MTT solution (20 *μ*L, 5 mg/mL) was added into each well. After 4 h of incubation, the culture medium in each well was abandoned and DMSO (200 *μ*L) was added to each well. The absorbance at 492 nm was measured using a microplate reader.

As controls, the biological stability and dark cytotoxicity of GNCs-ICG/Tf, as well as the dark cytotoxicity of GNCs-Pt and GNCs (without the functionalization of Tf, ICG, and mPEG-SH), were also investigated.

### 4.11. Cellular Uptake

HepG2 cells were seeded on a pretreated coverslip (22 × 22 mm^2^) in culture dishes at density of 1 × 10^5^ and cultured overnight. After the culture medium was abandoned, GNCs-Pt-ICG/Tf (50 *μ*g/mL) was dispersed in DMEM and incubated with cells for additional 2 h. Then, the uninternalized nanoparticles were washed away with PBS solution (1 mL × 3 times). The uptake of GNCs-Pt-ICG/Tf by HepG2 cells was observed by a Nikon Eclipse Ni-U upright optical microscope. As controls, cell uptake of GNCs-Pt-ICG (without Tf) and GNCs-ICG/Tf (without PtNPs) was also investigated. To assess the specific recognition capability of GNCs-Pt-ICG/Tf, the cell uptake of GNCs-Pt-ICG/Tf and GNCs-Pt-ICG by NCTC1469 cells (a mouse fibroblast cell line) was investigated.

### 4.12. Enhanced Dual-Modal Phototherapy Effect

The synergistic enhanced photodynamic/thermal therapy effect of GNCs-Pt-ICG/Tf was examined by CLSM. Firstly, HepG2 cells were seeded in culture dishes at density of 8 × 10^4^ and cultured overnight. Then, the cells were treated with GNCs-Pt-ICG/Tf for 24 h. The working concentration of GNCs-Pt-ICG/Tf was 50 *μ*g/mL, which has been proved to be safe for living cells without 808 nm laser irradiation. After washing the residual nanoparticles with PBS and addition of the fresh culture medium, the cells were irradiated by 808 nm laser at 2.0 W/cm^2^ for 10 min and incubated for another 24 h. Subsequently, all cells were stained with Hoechst 33342 (300 *μ*L, 100x) and PI (500 *μ*L, 0.03 mM) to distinguish living and dead cells before CLSM imaging.

As controls, the cell viability was also determined by cultivating HepG2 cells with GNCs-Pt-ICG/Tf (50 *μ*g/mL) in dark, GNCs-Pt-Tf (50 *μ*g/mL) upon 808 nm laser irradiation (2 W/cm^2^), as well as free ICG (1.66 *μ*mol/L) upon 808 nm laser irradiation (2 W/cm^2^), respectively. And the corresponding cell mortality of HepG2 cells was calculated by the signal intensity ratio between PI channel and DAPI channel.

## Data Availability

All data needed of this study are available in the article and its Supplementary Information files.

## Abbreviations

JNMs: Janus nanomotors
GNC: Gold nanocup
PtNPs: Platinum nanoparticles
GNC-Pt: A nanozyme-powered cup-shaped nanomotor constituted of a GNC with PtNPs at the bottom
PbS NPs: Octahedral PbS nanoparticles
POD: Peroxidase
Tf: Human transferrin
TfR: Transferrin receptor
ICG: Indocyanine green
mPEG-SH: Methoxy polyethylene glycol thiol
GNC-Pt-ICG/Tf: GNC-Pt with the functionalization of Tf, ICG, and mPEG-SH
GNC-ICG/Tf: GNC with the functionalization of Tf, ICG, and mPEG-SH
ROS: Reactive oxygen species
^1^O_2_: Singlet oxygen
CTAB: Hexadecyl trimethyl ammonium bromide
TMB: 3,5,3′,5′-tetramethylbenzidine
oxTMB: Oxidized TMB
SOSG: Singlet oxygen sensor green
PBS: Phosphate buffer saline
DMEM: Dulbecco's Modified Eagle Medium
PS: Penicillin/streptomycin
FBS: Fetal bovine serum
MTT: 3-(4,5-Dimethylthiazol-2-yl)-2,5-diphenyltetrazolium bromide
SPT: Single-particle tracking
LSPR: Localized surface plasmon resonance
UV-vis: Ultraviolet-visible
SEM: Scanning electron microscopy
TEM: Transmission electron microscopy
HAADF-STEM: High-angle annular dark-field scanning transmission electron microscopy
FT-IR: Fourier transform infrared
MSD: Mean-squared displacement
NIR: Near-infrared
PTT: Photothermal therapy
PDT: Photodynamic therapy
CLSM: Confocal laser scanning microscopy.

## Supplementary Materials

Supplementary Materials Experimental section. Supplementary figures and tables. Figure S1: modulating the LSPR of GNCs. Figure S2: the typical polarization-dependent scattering response (circles) from individual nanomaterials. Figure S3: characterization of PbS NPs and GNCs. Figure S4: kinetics for POD-like activity of GNCs-Pt. Figure S5: the pH stability of POD-like activity stability of GNCs-Pt. Figure S6: the average diffusion areas of GNCs-Pt with different H_2_O_2_ concentrations. Figure S7: dependence of average velocity ($\bar{v}$) of GNCs-Pt with different H_2_O_2_ concentrations. Figure S8: the distributions of *D*_*e*_ of individual GNCs-Pt. Figure S9: diffusion behaviors of GNCs during 10 s with different H_2_O_2_ concentrations. Figure S10: MSD versus the time interval (∆*t*) of GNCs-Pt with different H_2_O_2_ concentrations. Figure S11: typical trajectories of GNCs-Pt with different H_2_O_2_ concentrations. Figure S12: the temporal heterogeneity of diffusion behaviors of individual GNC-Pt with different H_2_O_2_ concentrations. Figure S13: fitting the curves of TA-MSD versus the time interval (∆*t*) of individual GNC-Pt with H_2_O_2_ (10%). Figure S14: fitting the curves of TA-MSD versus the time interval (∆*t*) of individual GNC-Pt in the absence of H_2_O_2_. Figure S15: the temporal heterogeneity of diffusion behaviors for GNC-Pt in the presence of 10% H_2_O_2_ by using a moving time-window method. Figure S16: the temporal heterogeneity of diffusion behaviors for GNCs-Pt in the absence of H_2_O_2_ by using a moving time-window method. Figure S17: characterization of GNCs-Pt-ICG/Tf. Figure S18: the loading capacity of ICG. Figure S19: the ^1^O_2_ generation capability of free ICG and GNCs-ICG/Tf. Figure S20: the photothermal effect of GNCs-Pt-ICG/Tf and GNCs-ICG/Tf. Figure S21: stability of GNCs-Pt-ICG/Tf in different media. Figure S22: stability of CTAB-stabilized GNCs-Pt in different media. Figure S23: cell viability of HepG2 cells after incubating with different nanomaterials. Figure S24: stability of GNCs-ICG/Tf in different media. Figure S25: stability of CTAB-stabilized GNCs in different media. Figure S26: the motion behaviors of GNCs-Pt-ICG/Tf and GNCs-ICG/Tf on HepG2 cell membrane. Figure S27: uptake efficiency of different nanomaterials for living cells. Table S1: *K*_*m*_ and *V*_max_ of GNCs-Pt towards TMB and H_2_O_2_. Table S2: fitting the curves of EA-TA-MSD versus the time interval (∆*t*) of GNCs-Pt with different H_2_O_2_ concentrations. Table S3: FT-IR analysis of GNCs-Pt-ICG/Tf (mPEG-SH) and GNCs-ICG/Tf (mPEG-SH).
