# Supplementary material for: Nanozyme-Triggered Cascade Reactions from Cup-Shaped Nanomotors Promote Active Cellular Targeting
Source: Research (Wash D C). 2022 Jun 19;2022:9831012. doi: 10.34133/2022/9831012 (PMC9275069; doi:10.34133/2022/9831012)
Supplement: Supplementary Materials — Experimental section. Supplementary figures and tables. Figure S1: modulating the LSPR of GNCs. Figure S2: the typical polarization-dependent scattering response (circles) from individual nanomaterials. Figure S3: characterization of PbS NPs and GNCs. Figure S4: kinetics for POD-like activity of GNCs-Pt. Figure S5: the pH stability of POD-like activity stability of GNCs-Pt. Figure S6: the average diffusion areas of GNCs-Pt with different H2O2 concentrations. Figure S7: dependence of average velocity (v¯) of GNCs-Pt with different H2O2 concentrations. Figure S8: the distributions of De of individual GNCs-Pt. Figure S9: diffusion behaviors of GNCs during 10 s with different H2O2 concentrations. Figure S10: MSD versus the time interval (∆t) of GNCs-Pt with different H2O2 concentrations. Figure S11: typical trajectories of GNCs-Pt with different H2O2 concentrations. Figure S12: the temporal heterogeneity of diffusion behaviors of individual GNC-Pt with different H2O2 concentrations. Figure S13: fitting the curves of TA-MSD versus the time interval (∆t) of individual GNC-Pt with H2O2 (10%). Figure S14: fitting the curves of TA-MSD versus the time interval (∆t) of individual GNC-Pt in the absence of H2O2. Figure S15: the temporal heterogeneity of diffusion behaviors for GNC-Pt in the presence of 10% H2O2 by using a moving time-window method. Figure S16: the temporal heterogeneity of diffusion behaviors for GNCs-Pt in the absence of H2O2 by using a moving time-window method. Figure S17: characterization of GNCs-Pt-ICG/Tf. Figure S18: the loading capacity of ICG. Figure S19: the 1O2 generation capability of free ICG and GNCs-ICG/Tf. Figure S20: the photothermal effect of GNCs-Pt-ICG/Tf and GNCs-ICG/Tf. Figure S21: stability of GNCs-Pt-ICG/Tf in different media. Figure S22: stability of CTAB-stabilized GNCs-Pt in different media. Figure S23: cell viability of HepG2 cells after incubating with different nanomaterials. Figure S24: stability of GNCs-ICG/Tf in different media. F [file 9831012.f1.docx]

Supporting Information

Nanozyme-Triggered Cascade Reactions from Cup-Shaped Nanomotors Promote Active Cellular Targeting

Xin Wang,^1^ Zhongju Ye,^2^ Shen Lin,^1^ Lin Wei,^3^ and Lehui Xiao^1^*

^1^ State Key Laboratory of Medicinal Chemical Biology, Tianjin Key Laboratory of Biosensing and Molecular Recognition, College of Chemistry, Nankai University, Tianjin, 300071

^2^ College of Chemistry, Zhengzhou University, Zhengzhou, 450001

^3^ Key Laboratory of Chemical Biology & Traditional Chinese Medicine Research, Key Laboratory of Phytochemical R&D of Hunan Province, College of Chemistry and Chemical Engineering, Hunan Normal University, Changsha, 410082

Correspondence should be addressed to Lehui Xiao; lehuixiao@nankai.edu.cn.

**Table of Contents**

[**Experimental section** 2](#_Toc97998527)

[**1.** **The selective deposition mechanisms of Au on PbS NPs** 2](#_Toc97998528)

[**2.** **The kinetic study of the catalytic process** 2](#_Toc97998529)

[**3.** **Design and driving mechanism of GNCs-Pt** 2](#_Toc97998530)

[**3.1 Design of GNCs-Pt** 2](#_Toc97998531)

[**3.2 Driving mechanism of GNCs-Pt** 3](#_Toc97998532)

[**4.** **Self-propulsion diffusion behavior analysis** 3](#_Toc97998533)

[**4.1 Experimental design** 3](#_Toc97998534)

[**4.2 Analysis methods** 4](#_Toc97998535)

[**5.** **The preparation of Tris-HCl buffer** 5](#_Toc97998536)

[**6.** **The loading capacity of ICG** 5](#_Toc97998537)

[**Supplementary figures** 7](#_Toc97998538)

[**Supplementary tables** 35](#_Toc97998539)

[**Supplementary references** 38](#_Toc97998540)

**Experimental section**

1. **The selective deposition mechanisms of Au on PbS NPs**

The selective deposition mechanisms of Au on PbS NPs have been explored in the previous report [1]. Because the atoms at the vertices and edges of nanocrystals are under-coordinated, they generally have higher energies than those on the perfect outer facets. The nucleation of a second material therefore generally takes place preferentially at these sites. As PbS (-4.92 eV) has a higher Fermi level than Au (-5.1 eV), the electron transfer is conducted from PbS to Au. Because a single octahedral PbS NP has only a limited number of electrons, the electron transfer results all of the other vertices of the octahedral PbS NP electron-deficient. Therefore, Au nucleation on the other vertices is prevented, making the selective deposition of Au only on one vertex.

Importantly, it is inspired by this facile method that we *in situ* grow small PtNPs asymmetrically at the bottom of gold nanocup (GNC) to fabricate the nanozyme-powered cup-shaped nanomotor (GNC-Pt) for the first time. The Janus structure is conducive to generate asymmetric propulsion force to break Brownian motion, resulting in short-ranged directional diffusion, which facilitates broader diffusion areas and efficient recognition toward biological targets.

1. **The kinetic study of the catalytic process**

The kinetic study of the catalytic process was analyzed according to Michaelis-Menton equation. The Michaelis Menten constant ($K_{m}$) was calculated through the Lineweaver Burk plot:

$\frac{1}{v}=\frac{K_{m}}{V_{max}}\times\frac{1}{\left[ S \right]}+\frac{1}{V_{max}}$ (S1)

Where $v$ is the initial velocity, $K_{m}$ is the Michaelis constant, $V_{max}$ is the maximal reaction velocity, and $\left[ S \right]$ is the concentration of substrate.[2] $v$ can be obtained by the absorbance at 650 nm and the molar absorption coefficient of oxidation product of TMB (oxTMB, $\varepsilon_{oxTMB}=$39000/(cm⋅mol/L)).[3]

1. **Design and driving mechanism of GNCs-Pt**

**3.1 Design of GNCs-Pt**

The toxicity of the exhaust gas (such as H_2_, CO_2_, ammonia) of nanomotors to the human body is yet to be tackled for broader applications and future clinical translation.[4] To address this challenge, we designed GNCs-Pt-ICG/Tf nanomotors from the following two aspects: On the one hand, drive the nanomotors *via* self-electrophoresis rather than bubble-propelled. The *in situ* growth of PtNPs on GNC asymmetrically leads to the contact between Au and Pt. The electrochemical reaction generated on the Pt-Au system, propelling GNCs-Pt-ICG/Tf nanomotors by self-electrophoresis. On the other hand, utilize the generated O_2_ to improve the restriction of the hypoxia tumor microenvironment on PDT. Therefore, O_2_ generated on Pt anode is consumed in two ways: partial generated O_2_ could be reduced on Au cathode for propelling nanomotors, and the rest of O_2_ is released into the hypoxic tumor environment for enhanced PDT.

**3.2 Driving mechanism of GNCs-Pt**

The corresponding chemical reactions of H_2_O_2_ on the surface of GNCs-Pt are listed below (Equation. S2–S5):[5]

Anode (Pt): $H_{2}O_{2}\underset{\to}{Pt}2H^{+}+O_{2}+2e^{-}$ (S2)

Cathode (Au): $\frac{1}{2}O_{2}+2H^{+}+2e^{-}\underset{\to}{Au}H_{2}O$ (S3)

Cathode (Au): $H_{2}O_{2}+2H^{+}+2e^{-}\underset{\to}{Au}2H_{2}O$ (S4)

Overall (GNCs-Pt): $2H_{2}O_{2}\underset{\to}{GNCs-Pt}{2H}_{2}O+O_{2}$ (S5)

Since Pt and Au in our GNCs-Pt are directly contacted, the decomposition of H_2_O_2_ belongs to an electrochemical reaction, which can trigger the self-electrophoresis of GNCs-Pt (Figure 4a). Firstly, the electrochemical oxidation of H_2_O_2_ is catalyzed on the Pt anode where protons and O_2_ molecules are generated. The electrons transfer from the Pt anode to the Au cathode, which results in the asymmetric charge distribution on the surface of GNCs-Pt. Then, protons would flow from Pt anode to Au cathode along the surface of GNCs-Pt for charge balance. Therefore, GNCs-Pt move in the opposite direction, an effect similar to electrophoresis.[5] There are partial H_2_O_2_ and O_2_ reduced on Au cathode. However, as shown in the balanced chemical equation ${2H}_{2}O_{2}\underset{\to}{GNCs-Pt}{2H}_{2}O+O_{2}$, the rest of O_2_ is released into the surrounding hypoxic tumor environment, which holds great potential for enhanced PDT.

1. **Self-propulsion diffusion behavior analysis**

**4.1 Experimental design**

H_2_O_2_ concentration is important for the self-diffusion behavior of GNC-Pt. When the diffusion of GNCs-Pt is too fast, it is difficult to capture their long and continuous trajectories, which can be ascribed to the limitation of the focal length of the objective lens and the frame rate of the camera. However, when the diffusion of GNCs-Pt is too slow, the diffusion behaviors are easily disturbed or even covered by Brownian motion. One of the ways to address this issue is to increase the viscosity of the medium to slow down the diffusion velocity. This method has been reported, such as addition of 50% (v/v) glycerol or 5 wt% polyvinylpyrrolidone in water.[6-7] Therefore, the investigations on the enhanced diffusion of GNC-Pt were conducted in a 50% water-glycerol mixture at relatively high concentrations of H_2_O_2_.

On the other hand, studying the dependence of the self-propulsion capability of GNCs-Pt on the concentration of H_2_O_2_ is of great significance not only to regulate the self-propulsion behavior of GNCs-Pt, but also to their biological application. However, it is not enough to fully reveal this dependence by just limiting the concentration of H_2_O_2_ to a low level as *in vivo*. Therefore, the range of H_2_O_2_ concentrations in aqueous experiments was higher than that *in vivo*. The results demonstrate that, with the concentration of H_2_O_2_ increased from 0 to 10%, the diffusion area, velocity, and effective diffusion coefficient (D_𝑒_) of GNCs-Pt increased accordingly (Figures 4e, S6-S8).

Actually, in order to understand the diffusion behavior of individual nanomotor, the concentration of fuels in solution is usually higher than that *in vivo*, which has been reported before. For example, in the work “*Bio-inspired nitric-oxide-driven nanomotor*”, the movement behavior of individual HLA_10_ nanomotor was performed at broad concentration range (0, 5, 10, and 20%, respectively).[4] Then, the uptake behaviors of HLA_10_ nanomotor by MCF-7 cells were also investigated under different H_2_O_2_ concentration (with or without extra 0.002% H_2_O_2_ addition) by confocal laser scanning microscopy (CLSM).

The experiments designed in this work are comparable to that reported previously. We investigated cellular uptake of GNCs-Pt-ICG/Tf and GNCs-ICG/Tf by HepG2 cells with dark-field microscopy. As shown in Figures 7b,c and S27, and the corresponding discussions in the manuscript, the number of GNCs-Pt-ICG/Tf in HepG2 cells is significantly higher than that of GNCs-ICG/Tf, indicating that GNCs-Pt-ICG/Tf nanomotors could promote the cellular recognition and uptake. This also proves that GNCs-Pt-ICG/Tf did carry out self-propelled diffusion in cell system.

**4.2 Analysis methods**

The tracking trajectories (n=30 in each group) of GNCs-Pt were extracted through ImageJ from the obtained videos. Then, the time-averaged mean-squared displacement (TA-MSD, $\left\langle{\Delta r\left( \Delta t \right)}^{2} \right\rangle$), effective diffusion coefficient ($D_{e}$) and the anomalous exponent ($\alpha$) were calculated. For a nanomotor *j* diffusing in two-dimension (2D), whose position coordinates $\boldsymbol{r}_{\boldsymbol{j}}$= {$\boldsymbol{x}_{\boldsymbol{j}}$, $\boldsymbol{y}_{\boldsymbol{j}}$} are sampled at *N* discrete times *m*Δ*t*:

$TA-MSD(t_{lag}=m\Delta t)=\left\langle{\Delta r\left( \Delta t \right)}^{2} \right\rangle=\frac{1}{N-m}\sum_{i=1}^{N-m} \left[ \boldsymbol{r}_{\boldsymbol{j}}(t_{i}+m\Delta t)-\boldsymbol{r}_{\boldsymbol{j}}(t_{i}) \right]^{2}$ (S6)

$\mathrm{MSD}\left( \Delta t \right)=2dD_{e}{\Delta t}^{\alpha}$, ($d=2, for 2D analysis$) (S7)

where $r$ is the position of the GNC-Pt in the x–y plane, the $\Delta r$ is the displacement of the GNCs-Pt during the lag time $\Delta t$, the angle brackets manifest an averaged value over all trajectories. According to the value of $\alpha$, the representative diffusion models can be classified as sub-diffusion ($\alpha<1$), Brownian motion ($\alpha\approx1$), and super-diffusion ($\alpha>1$).[8] The velocities of the GNCs-Pt in a series of different concentrations of H_2_O_2_ (0, 1, 2, 3, 5, and 10%, v/v) were also determined.

To estimate the heterogeneity among individual GNCs-Pt, the ensemble-time-averaged MSD (EA-TA-MSD) and ensemble-averaged MSD (EA-MSD) versus the time interval (∆t) of GNCs-Pt were also calculated.[9] To increase the statistics, the EA-TA-MSD can be further averaged over the ensemble composed by $J$multiple trajectories:

$EA-TA-MSD\left( t_{lag}=m\Delta t \right)=\frac{1}{J}\frac{1}{N-m}\sum_{j=1}^{j} \sum_{i=1}^{N-m} \left[ \boldsymbol{r}_{\boldsymbol{j}}(t_{i}+m\Delta t)-\boldsymbol{r}_{\boldsymbol{j}}(t_{i}) \right]^{2}$ (S8)

The EA-MSD versus the time interval ($\Delta t$) can be calculated for any initial time $t_{i}$ as the following equation:

$EA-MSD\left( t_{lag}=m\Delta t \right)=\frac{1}{J}\sum_{j=1}^{J} \left[ \boldsymbol{r}_{\boldsymbol{j}}(t_{i}+m\Delta t)-\boldsymbol{r}_{\boldsymbol{j}}(t_{i}) \right]^{2}$ (S9)

To reveal the precise characteristics and temporal heterogeneous within a single trajectory, we further investigated the individual trajectories by a moving time-window method (for 1.0 s). Briefly, the typical trajectory of GNCs-Pt was divided into 10 pieces sequentially by a moving time-window of 1.0 s, and the MSD, $D_{e}$, and $\alpha$ was further calculated using above equations.

1. **The preparation of Tris-HCl buffer**

Given that Tf has an isoelectric point of 5.9, Tf can easily couple with NHS-PEG-SH at the weak alkaline condition.[10] Therefore, Tris-HCl buffer (pH 8.5, 10 mM) was prepared. Briefly, Tris-HCl (23.64 mg, 0.15 mmol) was firstly dissolved and diluted with DI water to 15 mL. Then, the pH value of mixture was adjusted with 0.1 M HCl to 8.5.

1. **The loading capacity of ICG**

The amount of loaded ICG was determined by the calibration curve of the ICG ($Abs. =0.103\cdot c+0.016, R^{2}=0.999$). Then, the absorbance of the modification system was collected by a UV-Vis spectroscopy before and after ICG loaded. The loading efficiency was calculated through the equation S10:

Loading efficiency =$\frac{\left( \boldsymbol{m}_{\boldsymbol{ICG-before}}\boldsymbol{-}\boldsymbol{m}_{\boldsymbol{ICG-after}} \right)}{\boldsymbol{m}_{\boldsymbol{GNCs-Pt}}}$ (S10)

Where the $m_{ICG-before}$ is the amount of ICG in the stock solution before the GNCs-Pt loaded, $m_{ICG-after}$ is the residual amount of ICG after the GNCs-Pt loading. According to the Beer's Law, the amount of ICG loaded on the GNCs-Pt ($m_{ICG-before}$*,* $m_{ICG-after}$) was direct proportion to the UV-Vis absorbance (${Abs}_{ICG-before}$*,* ${Abs}_{ICG-after}$).

**Supplementary figures**

**
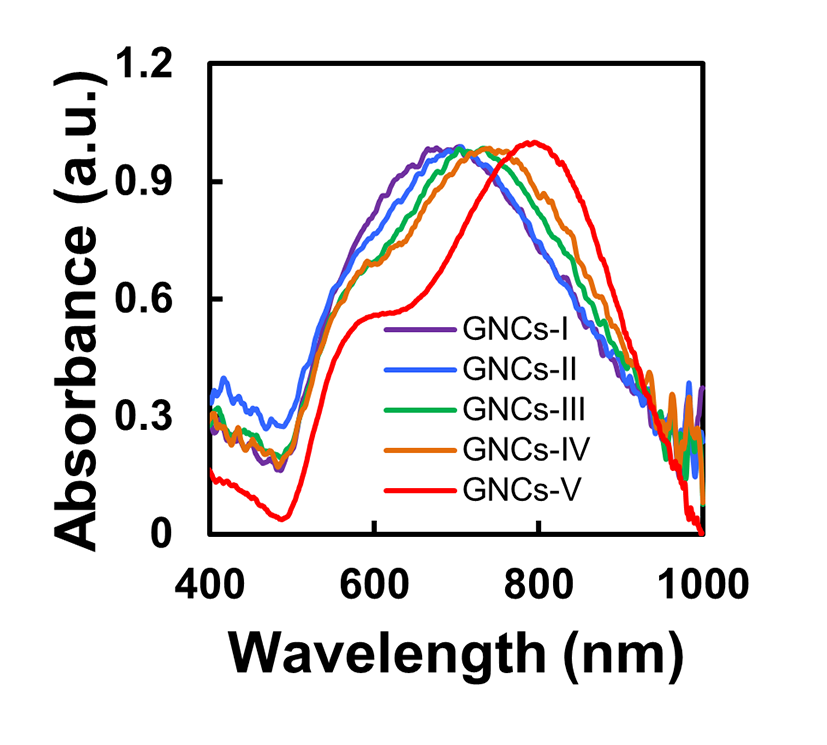
**

**Figure S1.** Modulating the localized surface plasmon resonance (LSPR) of GNCs. Normalized UV-vis spectra of GNCs synthesized with different concentrations of PbS NPs (I, II, III, IV, and V).

As shown in Figure S1, GNCs possess one major resonance peak and a shoulder at the higher-energy side. With the decrease of the PbS NPs concentrations, the major resonance peak red-shifts gradually and the shoulder resonance becomes clearer. Finally, the conditions for GNCs with the surface plasmon resonance (SPR) band in NIR range were optimized.


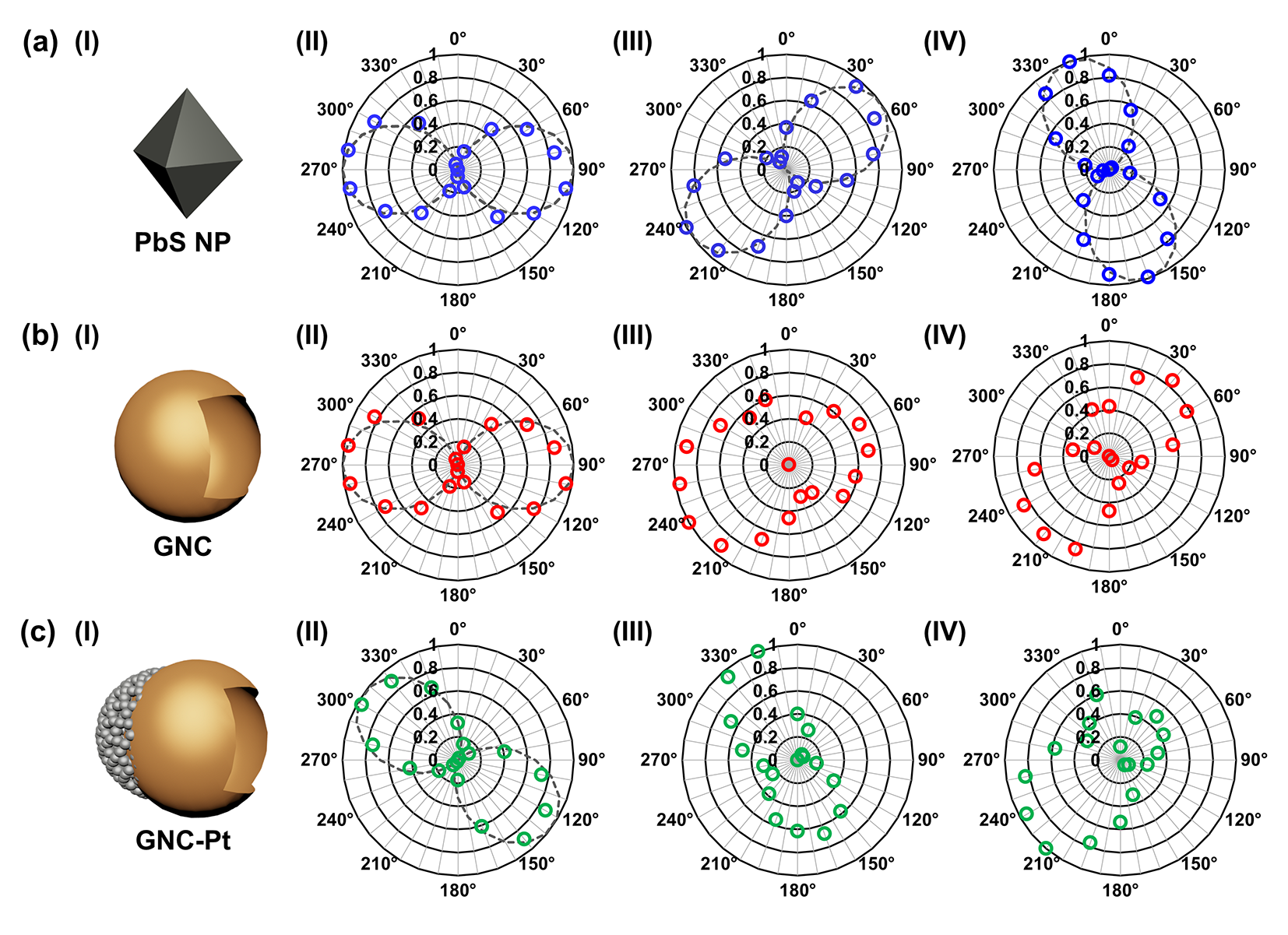


**Figure S2.** The typical polarization-dependent scattering response (circles) from individual (a) PbS NP, (b) GNC, and (c) GNC-Pt as a function of the angle relative to the optical axis of the polarizer. The gray dashed lines are the fitted curves based on the relationship of I∝cos^2^(θ).

For PbS NPs, the symmetry of the polarization-dependent scattering pattern is irrelevant to their orientation, indicating the symmetrical structure of PbS NPs. While for both of GNCs and GNCs-Pt, the symmetry of the polarization-dependent scattering pattern is orientation-dependent, suggesting the asymmetric structure and random orientation of GNCs and GNCs-Pt on the glass slide.


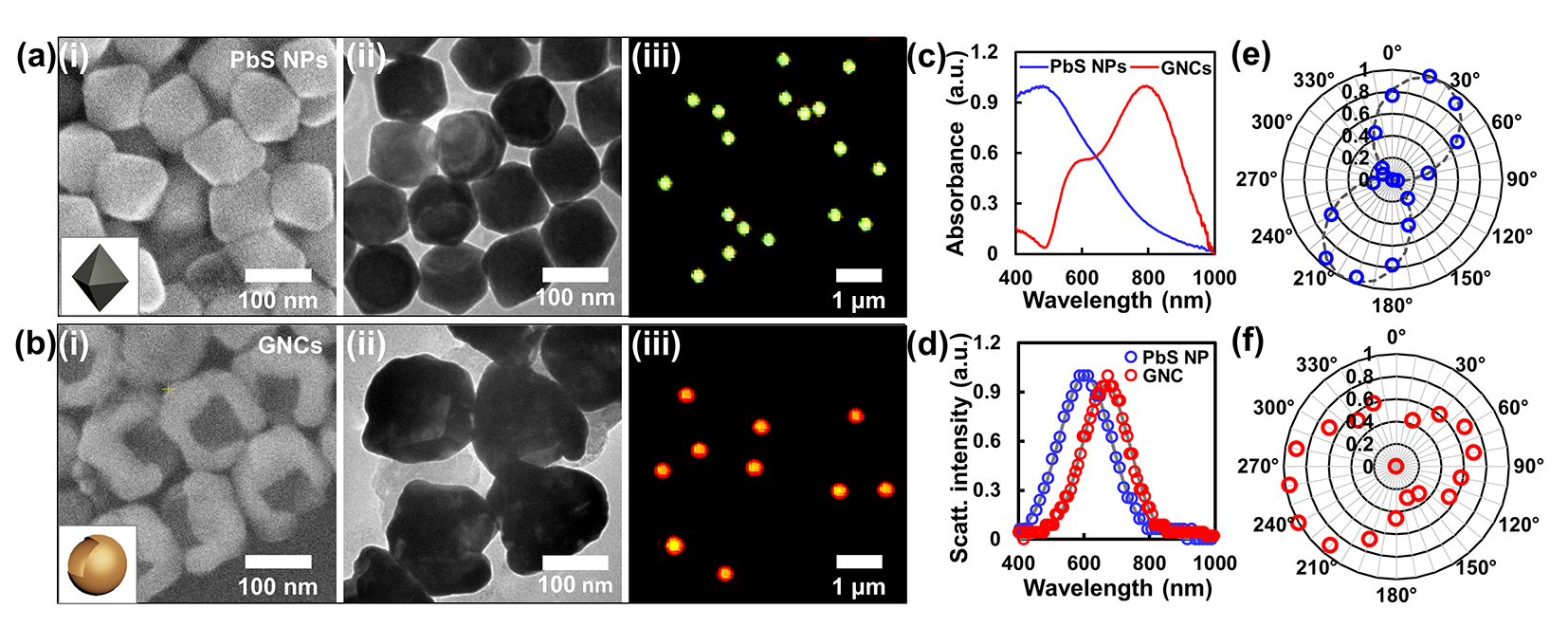


**Figure S3.** Characterization of PbS NPs and GNCs. (a) (i-iii) SEM, TEM, and dark-field optical microscopic images of PbS NPs (inset: schematic diagram of PbS NP). (b) (i-iii) SEM, TEM, and dark-field optical microscopic images of GNCs (inset: schematic diagram of GNC). (c) UV-vis spectra of PbS NPs and GNCs. (d) Single-particle scattering spectra of individual PbS NP and GNC. The gray line is the fitted curve based on Gaussian function. The polarization-dependent scattering response from individual (e) PbS NP and (f) GNC as a function of the angle relative to the optical axis of the polarizer. The gray dashed line in Figure (e) is the fitted curve based on the relationship of $I\propto{cos}^{2}\left( \theta\right)$.

The SEM and TEM images indicate the well-defined octahedral structure of PbS NPs and the cup-shaped structure of GNCs (Figures S3a and b). The well-defined yellowish-green and orange in color dark-field microscopic images as well as the evenly distributed scattering signals confirm the good monodispersity of PbS NPs and GNCs, respectively. PbS NPs exhibit unobvious absorption peak in the 400-1000 nm region. While the obvious major resonance peak and a shoulder at the higher-energy side further indicate the asymmetric structure of GNCs (Figure S3c). Furtherly, the symmetry of the polarization-dependent scattering pattern of individual PbS NP is irrelevant to its orientation (Figure S3e).[11] In contrast, the orientation-dependent dipole pattens in polarization modulation experiments verify the asymmetric structure and random orientation of GNCs on the glass slide (Figure S3f).


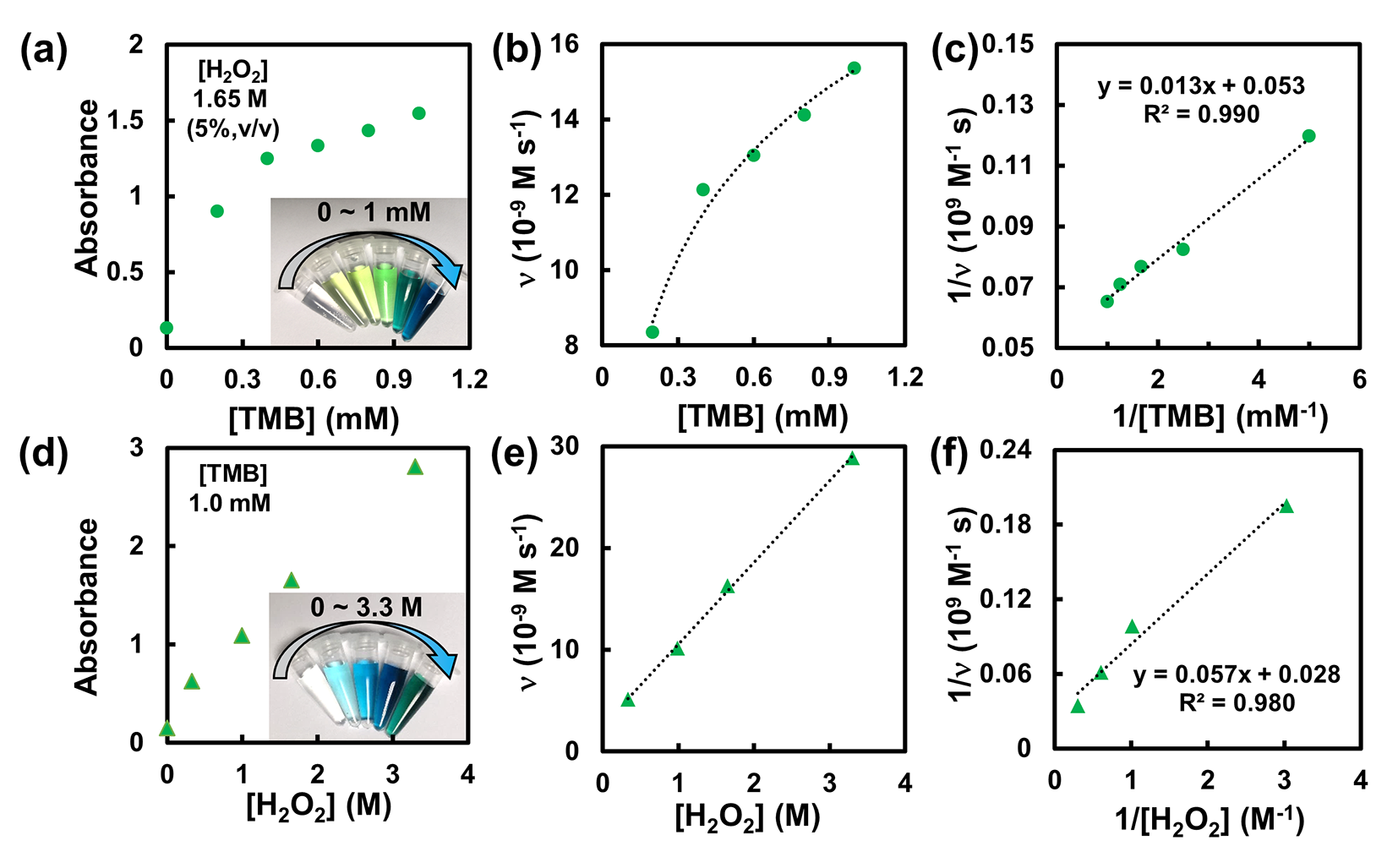


**Figure S4.** Kinetics for POD-like activity of GNCs-Pt. Absorbance at 650 nm, Michaelis-Menten curves, and Lineweaver-Burk plots for (a-c) various concentrations of TMB (0, 0.2, 0.4, 0.6, 0.8, and 1.0 mM) while H_2_O_2_ concentration fixed at 1.65 M, and (d-f) various concentrations of H_2_O_2_ (0, 0.33, 0.99, 1.65, and 3.3 M) while TMB concentration fixed at 1.0 mM. Inset: optical images of the oxidized TMB (oxTMB) produced under different catalytic conditions for 30 min.

As shown in Figure S4e, the maximal reaction velocity ($V_{max}$) of GNCs-Pt in the presence of H_2_O_2_ (10%) is more than 11-fold faster than that of GNCs-Pt in the absence of H_2_O_2_. The Michaelis-Menten constant ($K_{m}$) is calculated to be 0.25 mM for TMB substrate and 2.05 × 10^3^ mM for H_2_O_2_ substrate. These results demonstrate that GNCs-Pt possess POD-like activity, which provides an essential prerequisite for self-propulsion by utilizing the overexpressed endogenous H_2_O_2_ in tumor microenvironment.


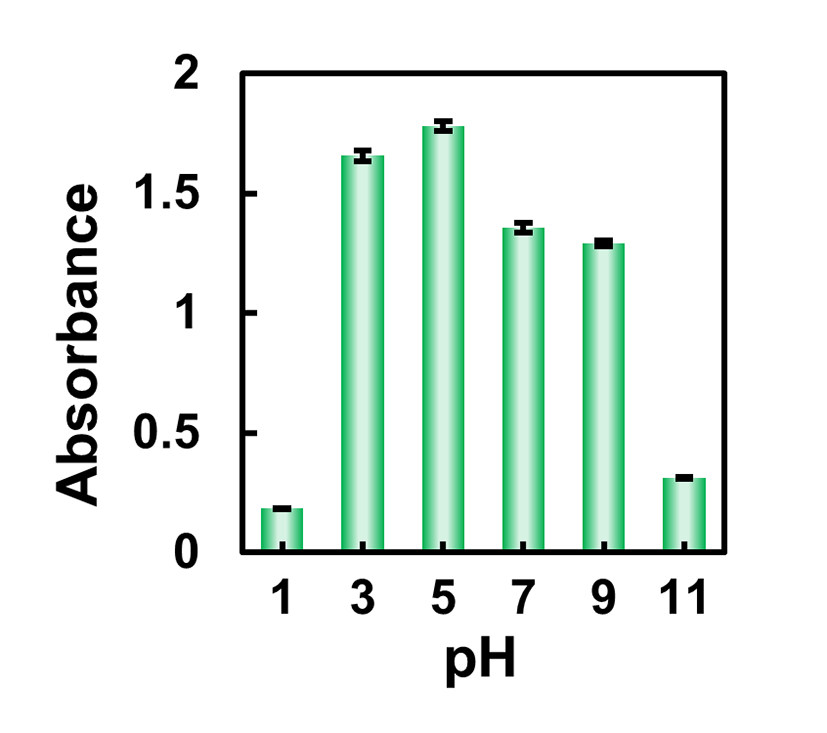


**Figure S5.** The pH stability of POD-like activity stability of GNCs-Pt. POD-like activity stability of GNCs-Pt in solutions with pH values in the range from 1 to 11, respectively.


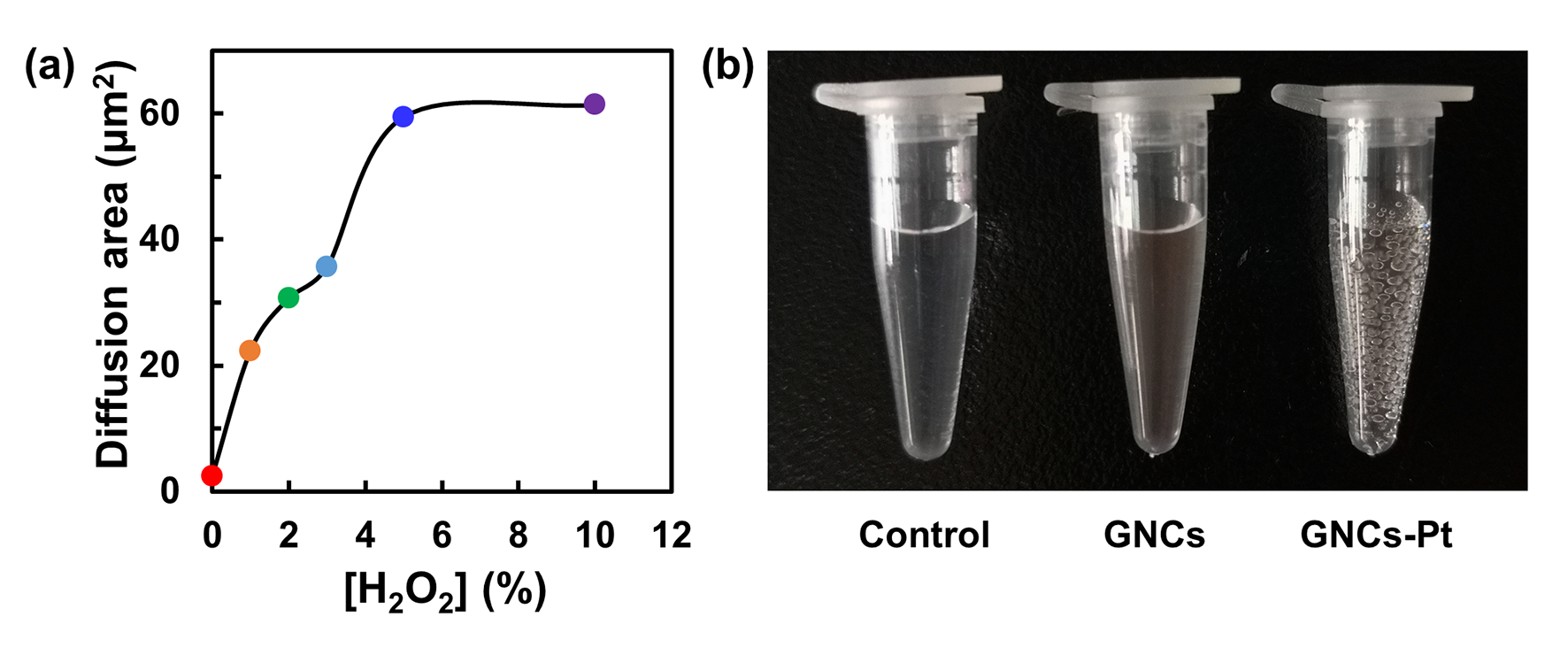


**Figure S6.** (a) The average diffusion areas of GNCs-Pt with different H_2_O_2_ concentrations (0, 1, 2, 3, 5, and 10%) during 10 s. (b) Optical images of GNCs and GNCs-Pt dispersed in H_2_O_2_ for 30 min.

The optical images of GNCs and GNCs-Pt dispersed in H_2_O_2_ for 30 min directly reflect the catalytic decomposition of H_2_O_2_ by GNCs-Pt.


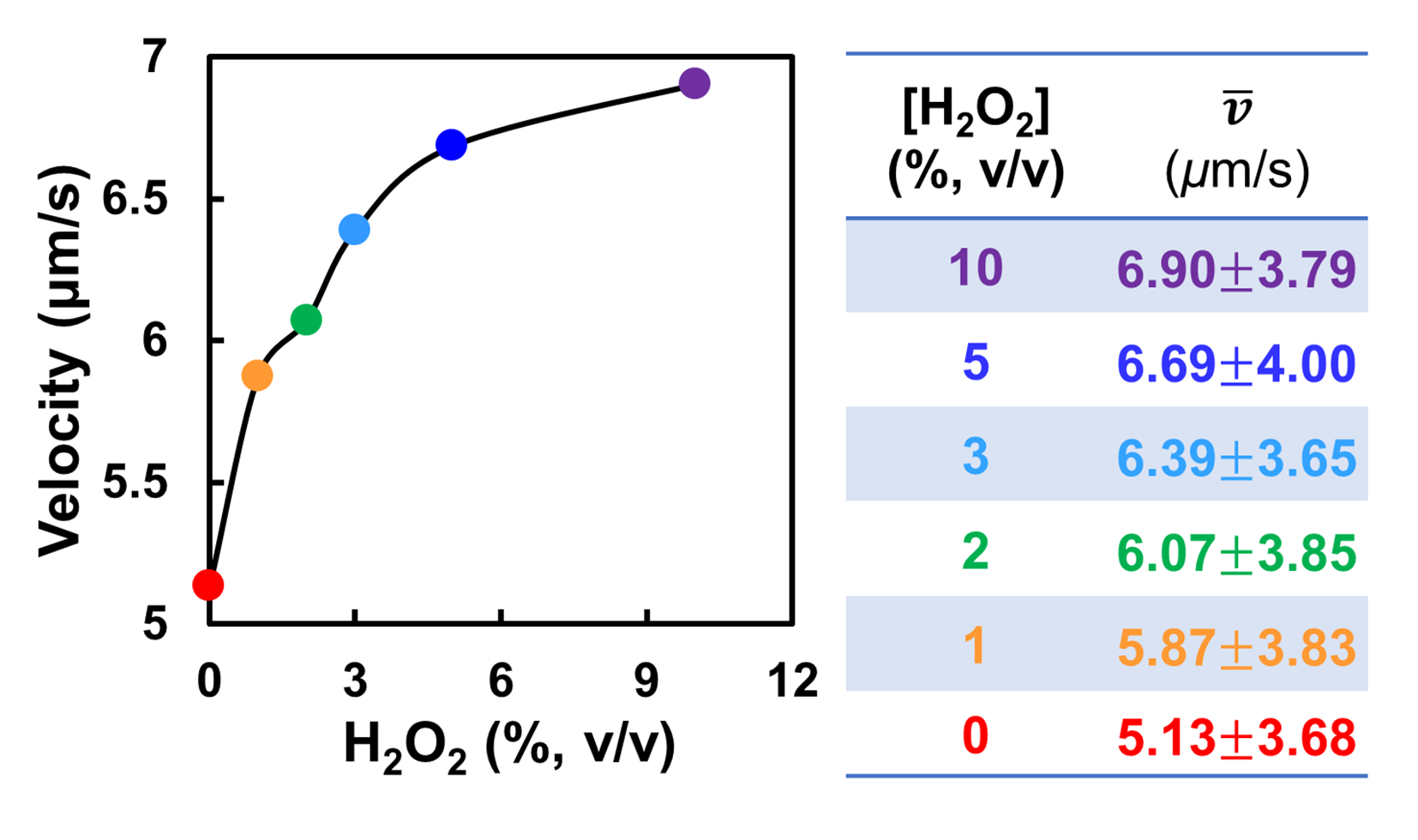


**Figure S7.** Dependence of average velocity ($\bar{v}$) of GNCs-Pt with different H_2_O_2_ concentrations (0, 1, 2, 3, 5, and 10%).


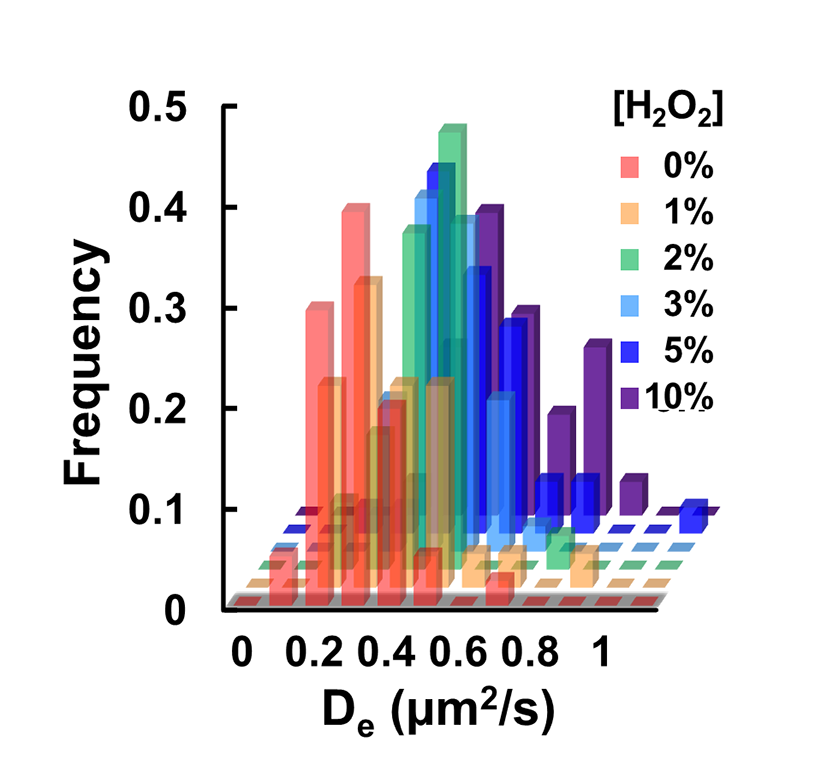


**Figure S8.** The distributions of $D_{e}$ of individual GNCs-Pt (n=30 in each group) obtained by analyzing TA-MSD with different H_2_O_2_ concentrations (0, 1, 2, 3, 5, and 10%).


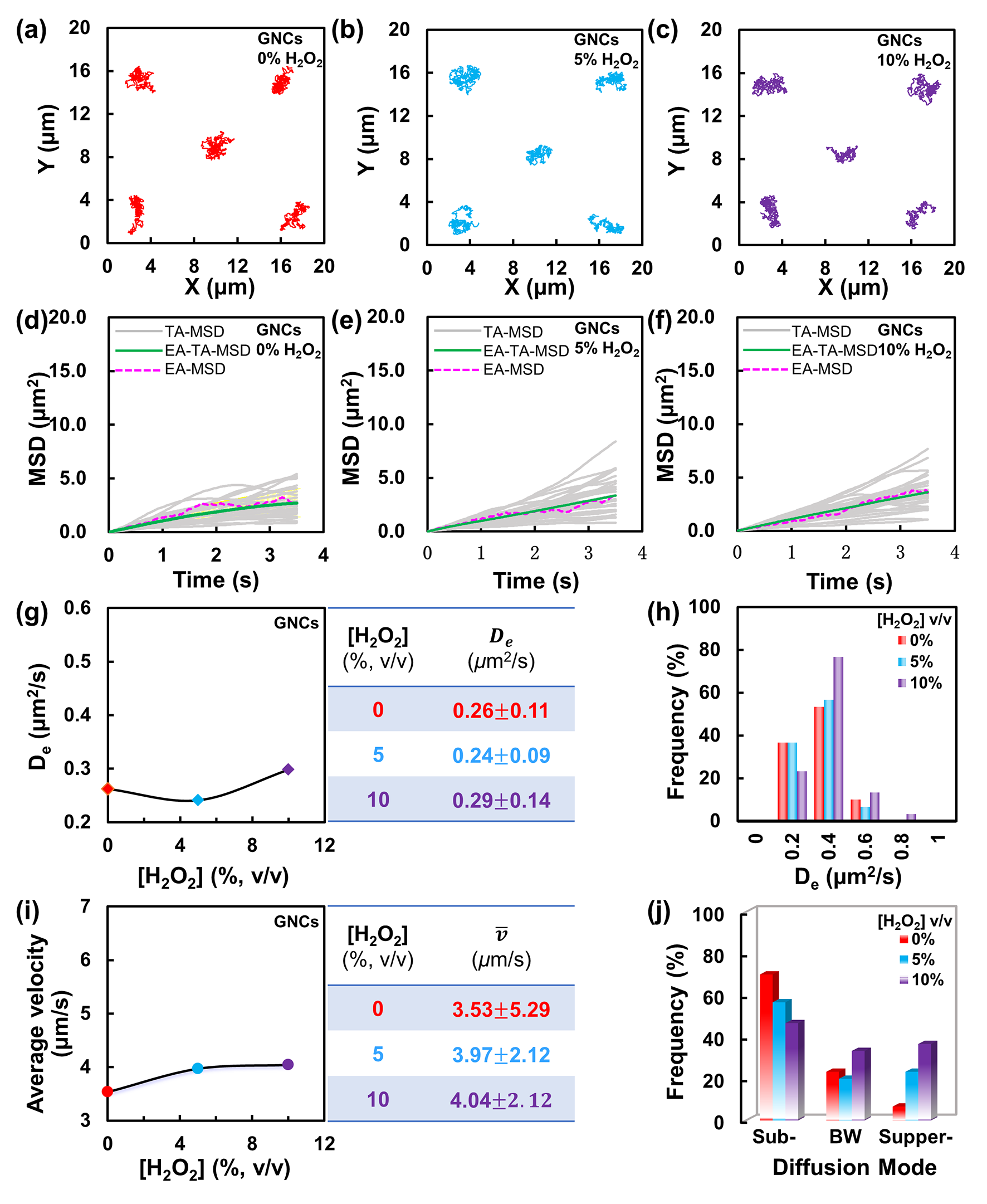


**Figure S9.** Diffusion behaviors of GNCs during 10 s with different H_2_O_2_ concentrations (0, 5, and 10%). (a-c) Representative trajectories, and (d-f) MSD of GNCs at different H_2_O_2_ concentrations. The solid lines (gray) represent the TA-MSD curves of individual trajectories, respectively. The thick lines (green) represent the EA-TA-MSD. The dashed curves (red) denote the EA-MSD calculated from all individual trajectories. (g, i) Dependence of $D_{e}$ and average velocity of GNCs with different H_2_O_2_ concentrations. (h, j) The distributions of $D_{e}$ and diffusion modes (including sub-diffusion, Brownian motion (BW), and super-diffusion) of GNCs-Pt with different H_2_O_2_ concentrations.

No significant differences were observed in the trajectories, EA-TA-MSD, $D_{e}$, and average velocity of GNCs with different H_2_O_2_ concentrations (0, 5, and 10%) because of the negligible POD-like activity of GNCs. These observations agree well with the results from POD-like activity tests.


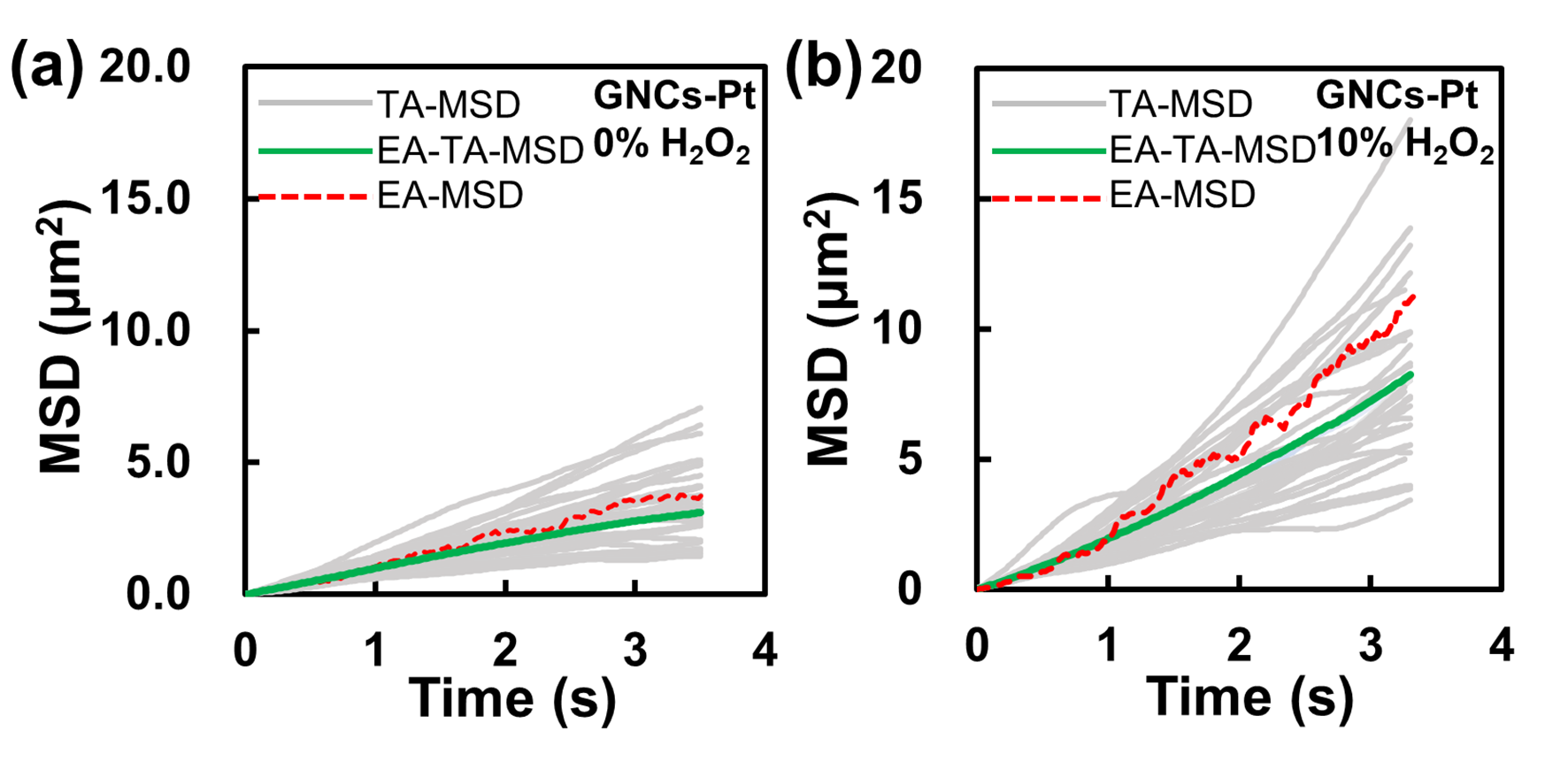


**Figure S10.** MSD versus the time interval (∆t) of GNCs-Pt in the (a) absence and (b) presence of H_2_O_2_ (10%). The solid lines (gray) represent the TA-MSD of individual trajectories, respectively. The thick lines (green) represent the EA-TA-MSD. The dashed curves (red) denote the EA-MSD calculated from all individual trajectories.

Whether or not in the presence of H_2_O_2_, EA-TA-MSD is inconsistent with TA-MSD and EA-MSD. These results indicate that not only the huge heterogeneity exists among individual GNCs-Pt, but also the various diffusion patterns exist at different time for the same GNC-Pt.


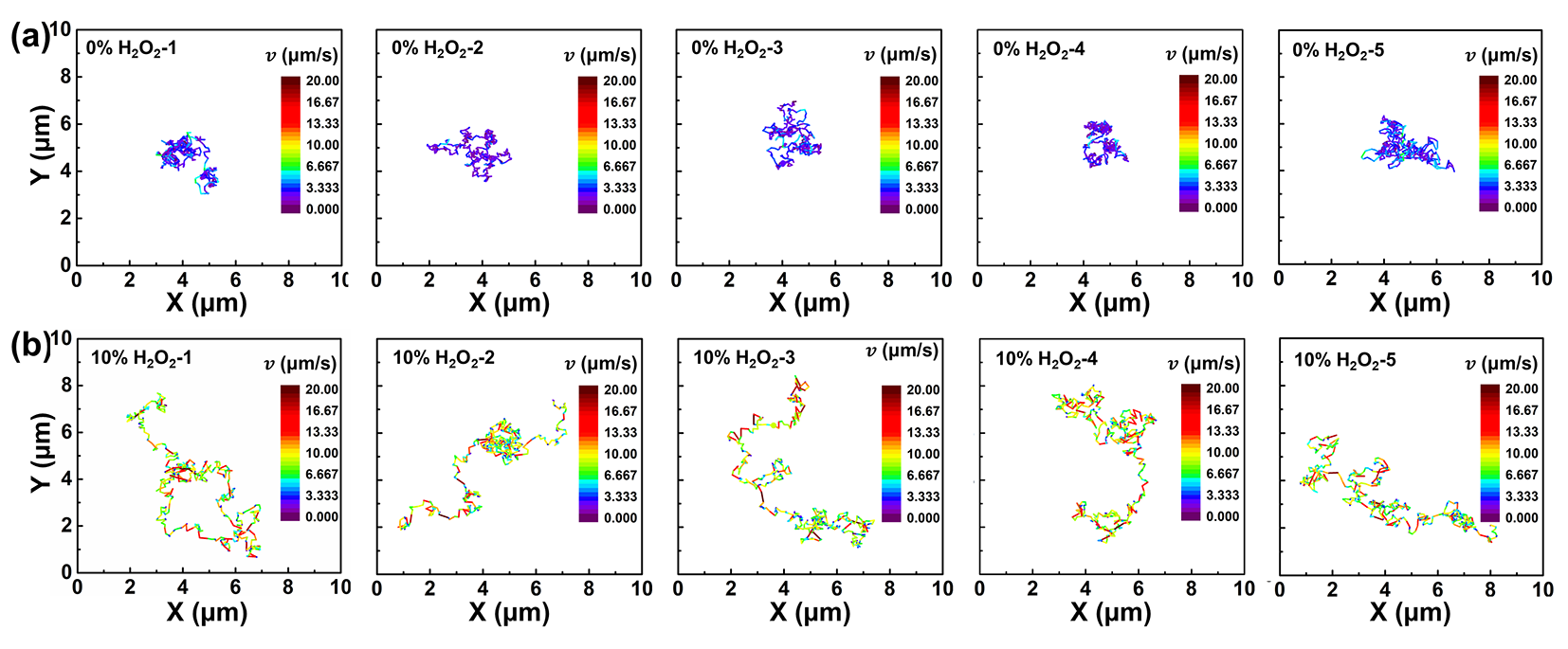


**Figure S11.** Typical trajectories of GNCs-Pt in the (a) absence and (b) presence of H_2_O_2_ (10%) by the color-coded speed in 10 s. The color bar from purple to deep red represents the instantaneous velocity ($v$) from 0 to 20 *μ*m/s.


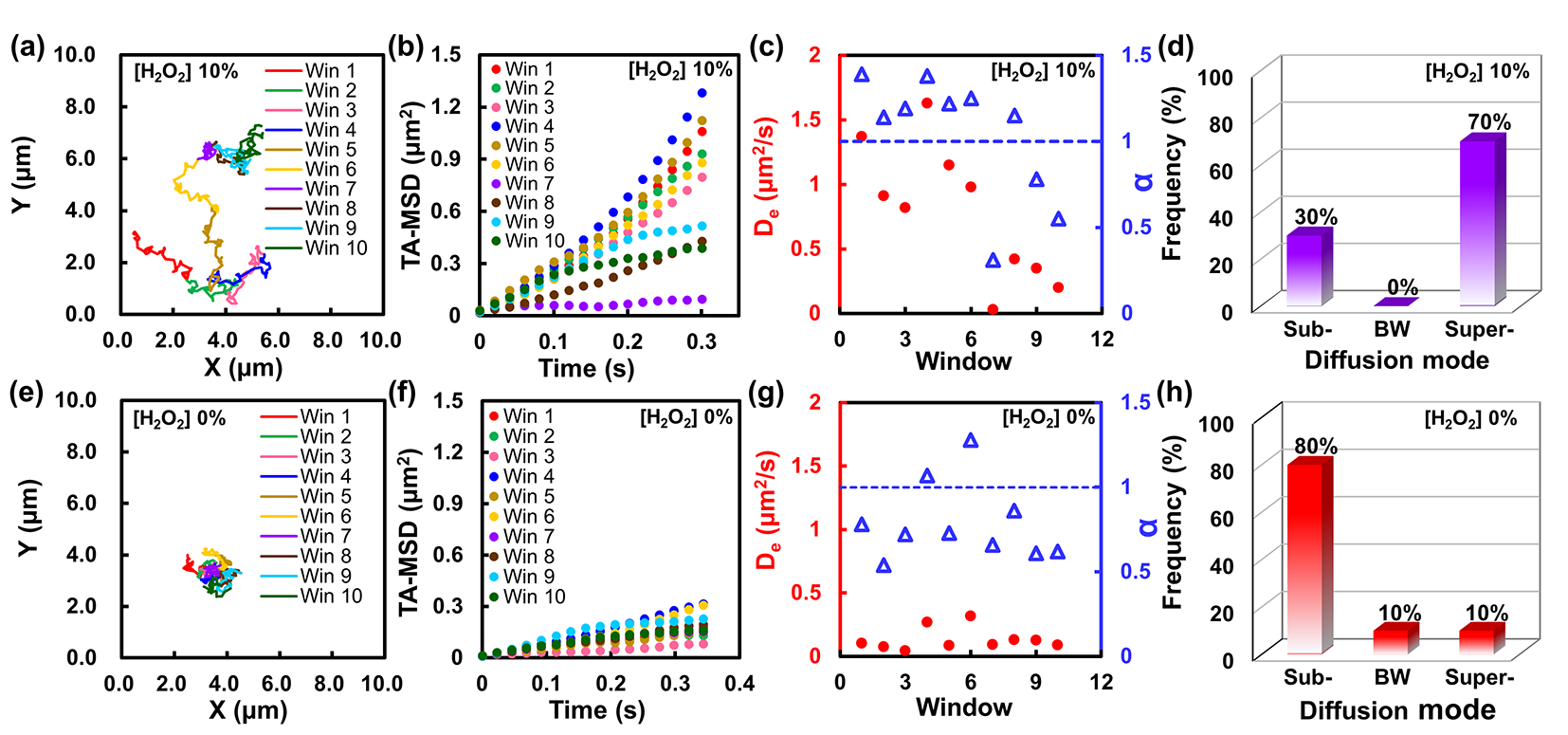


**Figure S12.** The temporal heterogeneity of diffusion behaviors of individual GNC-Pt in the presence (a-d) and absence (e-h) of H_2_O_2_ (10%) by a moving time-window method (for 1.0 s). (a,e) Typical trajectories (in 10 s), (b,f) TA-MSD, (c,g) $D_{e}$ and anomalous exponent (𝛼) of individual GNC-Pt during each moving window. (d,h) The distributions of the diffusion modes (including sub-diffusion, Brownian motion (BW), and super-diffusion) of individual GNC-Pt.


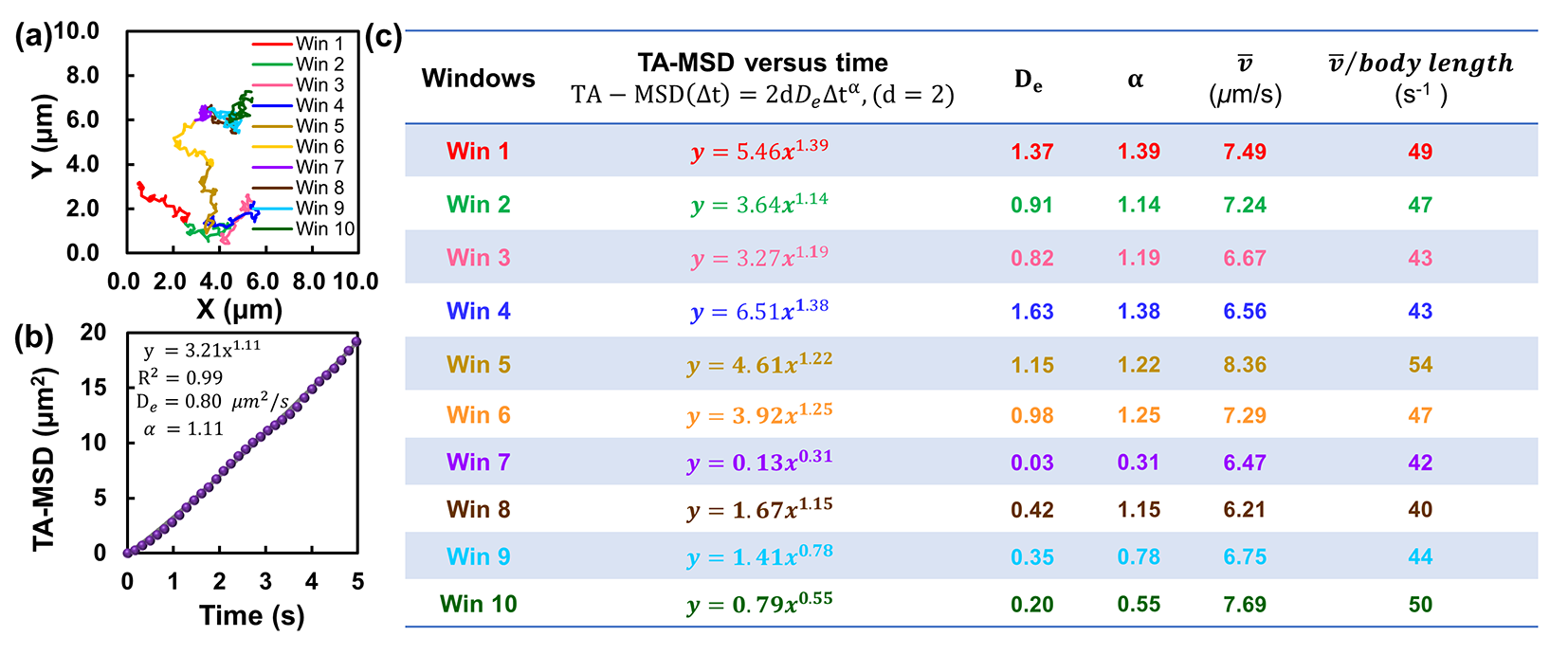


**Figure S13.** Fitting the curves of TA-MSD versus the time interval (∆t) of individual GNC-Pt with H_2_O_2_ (10%). Equation TA-$\mathrm{MSD}\left( \Delta t \right)=2dD_{e}{\Delta t}^{\alpha}, d=2$ is employed to estimate the effective diffusion coefficient ($D_{e}$) and anomalous exponent ($\alpha$). (a) Trajectory, (b) TA-MSD, (c) D_e_, α, and average velocity ($\bar{v}$) of typical GNC-Pt in each time window.


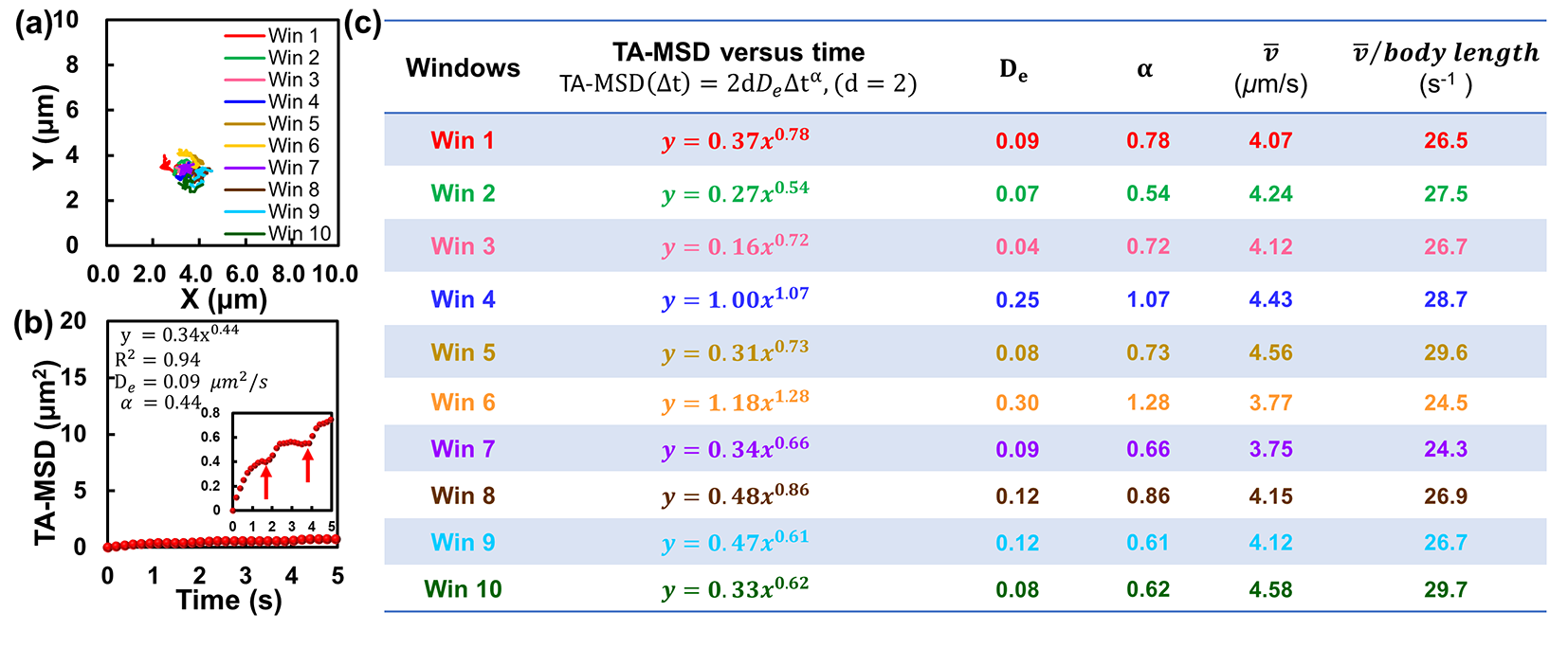


**Figure S14.** Fitting the curves of TA-MSD versus the time interval (∆t) of individual GNC-Pt in the absence of H_2_O_2_. Equation TA-$\mathrm{MSD}\left( \Delta t \right)=2dD_{e}{\Delta t}^{\alpha}, d=2$ is employed to estimate the effective diffusion coefficient ($D_{e}$) and anomalous exponent ($\alpha$). (a) Trajectory, (b) TA-MSD, (c) D_e_, α, and average velocity ($\bar{v}$) of typical GNC-Pt in each time window.

According to the distributions of $D_{e}$ and $\alpha$ of individual GNC-Pt in 10% H_2_O_2_ (v/v), super-diffusion (70%) dominated over Brownian motion (0%) and sub-diffusion (30%) (Figures S12ad and S13). Surprisingly, in the absence of H_2_O_2_, GNC-Pt was dominated by sub-diffusion (80%), while Brownian motion (10%) and super-diffusion (10%) with lower probabilities did exist and alternate randomly (Figures S12e-h and S14).


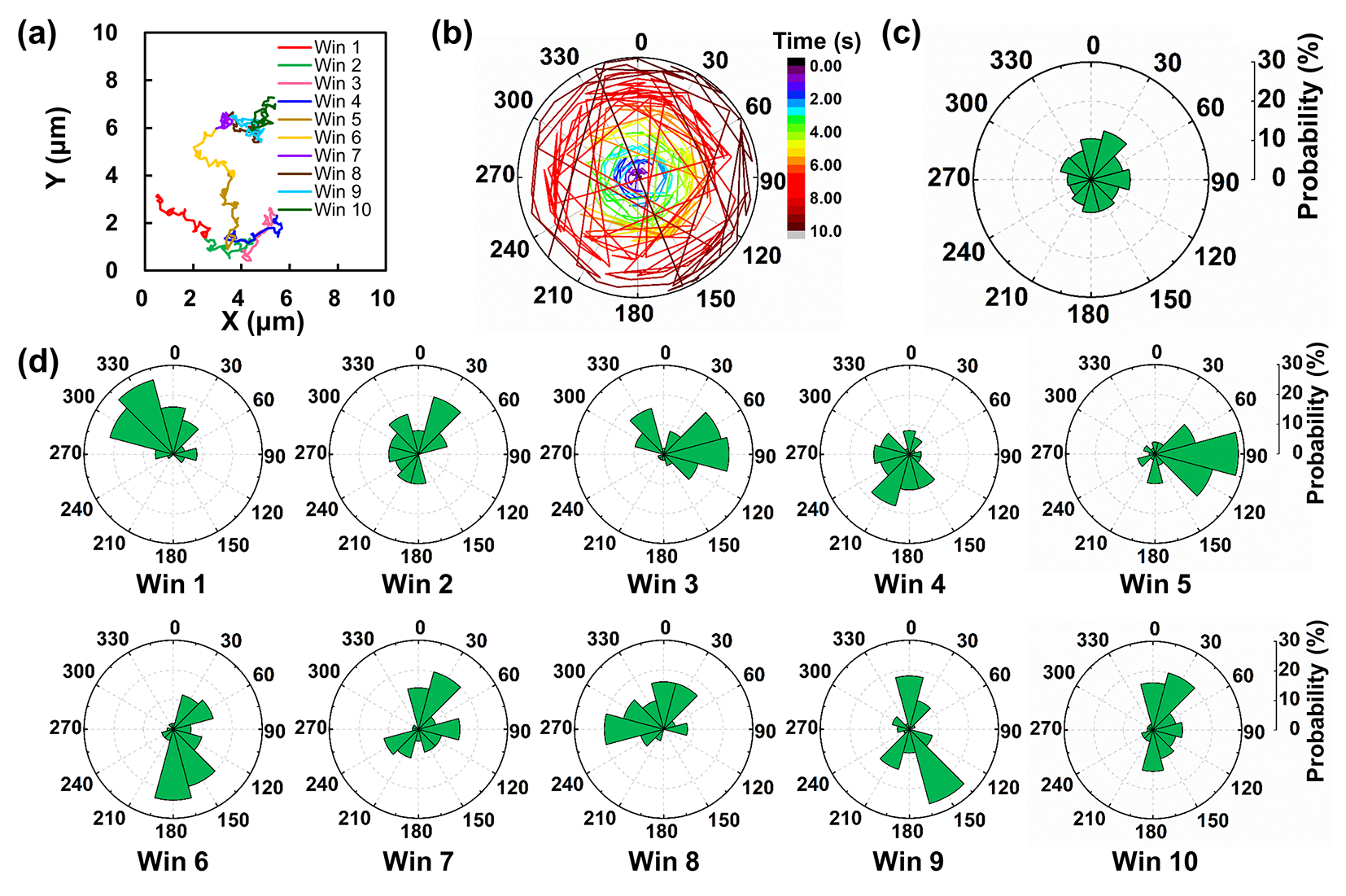


**Figure S15.** The temporal heterogeneity of diffusion behaviors for GNC-Pt in the presence of 10% H_2_O_2_ (v/v) by using a moving time-window mothed (1.0 s). (a) Trajectory. (b) Azimuthal angle (φ) with the color-coded time during 10 s. (c,d) Distributions of φ during 10 s as well as in each time window.


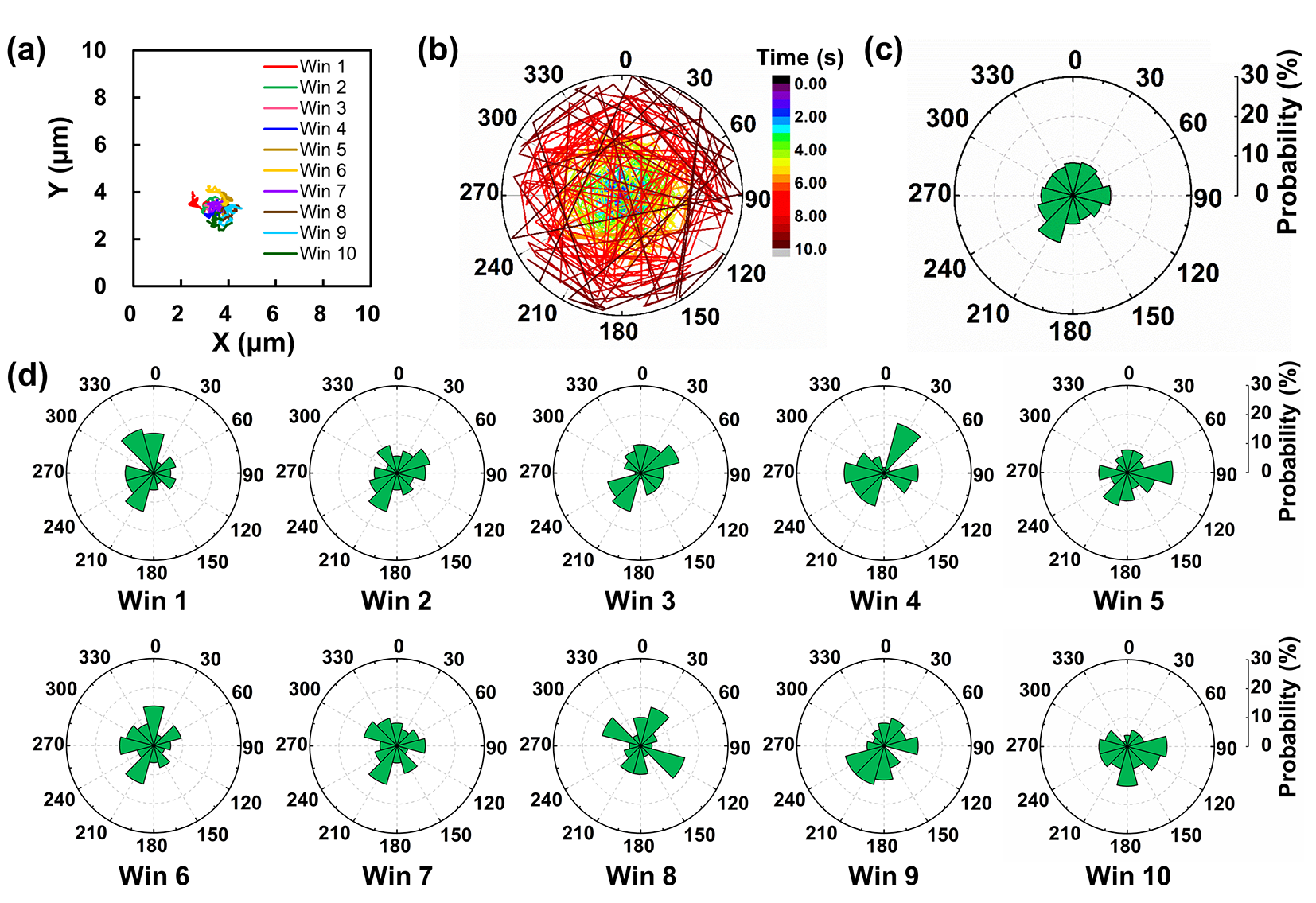


**Figure S16.** The temporal heterogeneity of diffusion behaviors for GNCs-Pt in the absence of H_2_O_2_ by using a moving window of 1.0 s. (a) Trajectory. (b) Azimuthal angle (φ) with the color-coded time during 10 s. (c,d) Distributions of azimuthal $\varphi$ during 10 s as well as in each time windows.


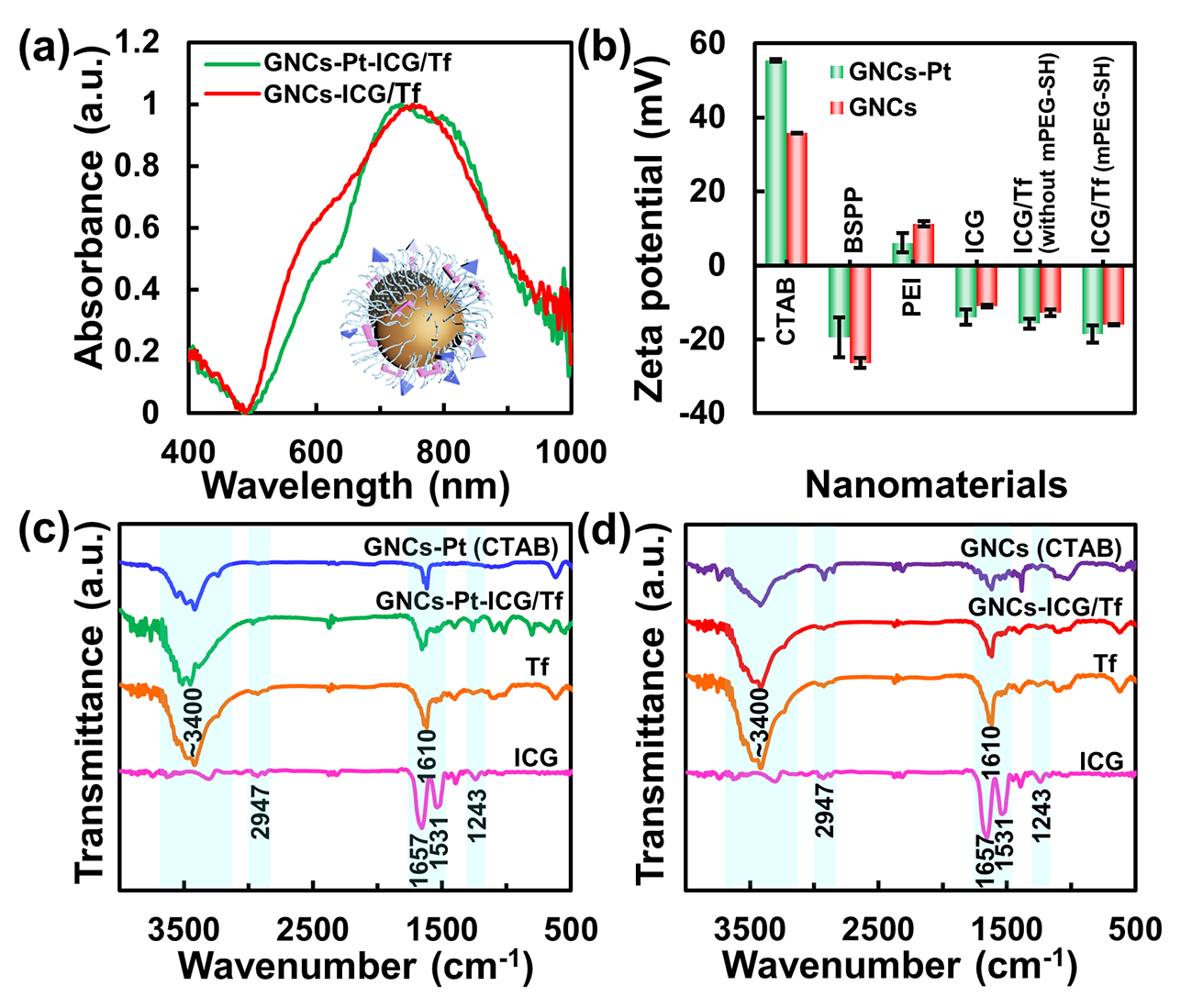


**Figure S17.** Characterization of GNCs-Pt-ICG/Tf. (a) UV-vis spectra of GNCs-ICG/Tf and GNCs-Pt-ICG/Tf, respectively (inset: schematic diagram of GNC-Pt-ICG/Tf). (b) Zeta potentials of GNCs and GNCs-Pt stabilized with CTAB, and modified with BSPP, PEI, ICG, as well as ICG and Tf (without or with mPEG-SH), respectively. (c,d) FT-IR characteristic of GNCs-Pt-ICG/Tf (mPEG-SH) and GNCs-ICG/Tf (mPEG-SH), respectively.

As shown in Figure S17a, a shoulder peak at ~800 nm can be assigned to ICG in the UV-vis spectrum of GNCs-Pt-ICG/Tf.[12] The overlap between the excitation wavelength of ICG and the SPR of GNCs-Pt provides a good opportunity to achieve synergistic PDT/PTT upon 808 nm laser irradiation. In addition, the zeta potential of GNCs-Pt-ICG/Tf reversed from positive (+55.4 mV of GNCs-Pt) to negative (-18.6 mV), indicating the substitution of CTAB and the modification of ICG and Tf (Figure S17b). The successful modification was further confirmed by the characteristic peaks of ICG, Tf, and PEG in the FT-IR spectra of GNCs-Pt-ICG/Tf (Figures S17c and d). Typical peaks at ~1243, ~1531, ~1657, and ~2947 cm^-1^ can be attributed to the –S=O stretching, C=C stretching of naphthalene skeleton, C=C stretching of alkene, and C–H stretching vibration of ICG, respectively. The peak at ~1610 cm^-1^ arises from C=O stretching vibrations of amide in Tf.[13] In addition, the characteristic peaks at ~1016 and ~1099 cm^-1^ can be assigned to the stretching vibrations of C-O of mPEG. And the peak at ~1411 cm^-1^ can also be attributed to the -CH_2_- bending vibrations of mPEG.[14] Therefore, the above experimental results clearly verify the successful decoration of ICG and Tf on GNCs-Pt. The successful decorations of ICG, Tf, and PEG on GNCs were also proved by similar characterizations.


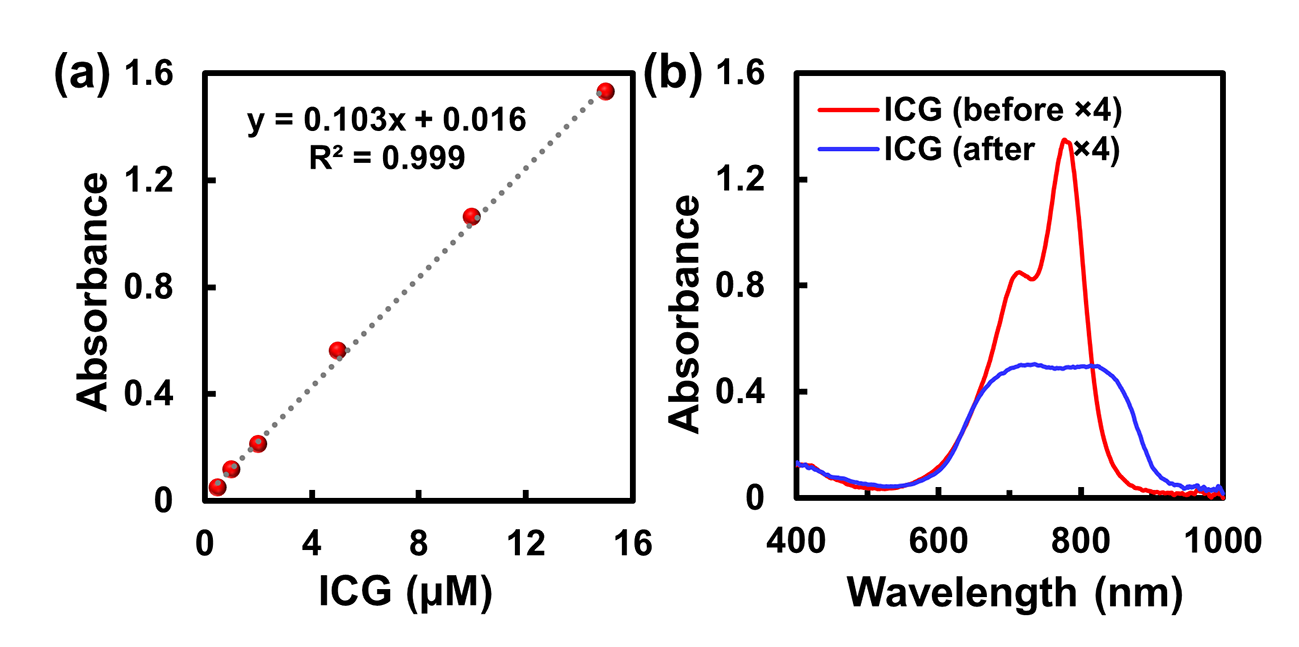


**Figure S18.** The loading capacity of ICG. (a) Calibration curve between the concentration and the absorbance of ICG. The standard curve equation of ICG solution is ${Abs.}_{\lambda=780 nm}=0.103 c_{ICG}+0.016, R^{2}=0.999$. (b) UV-vis spectra of ICG solution before and after loaded by the GNCs-Pt (the solution was diluted four folds with DI water before detection).

According to the Lambert-Beer Law, the loading capacity of ICG is calculated to be 25.97 mg (33.51 *μ*mol) for 1.0 g GNCs-Pt.


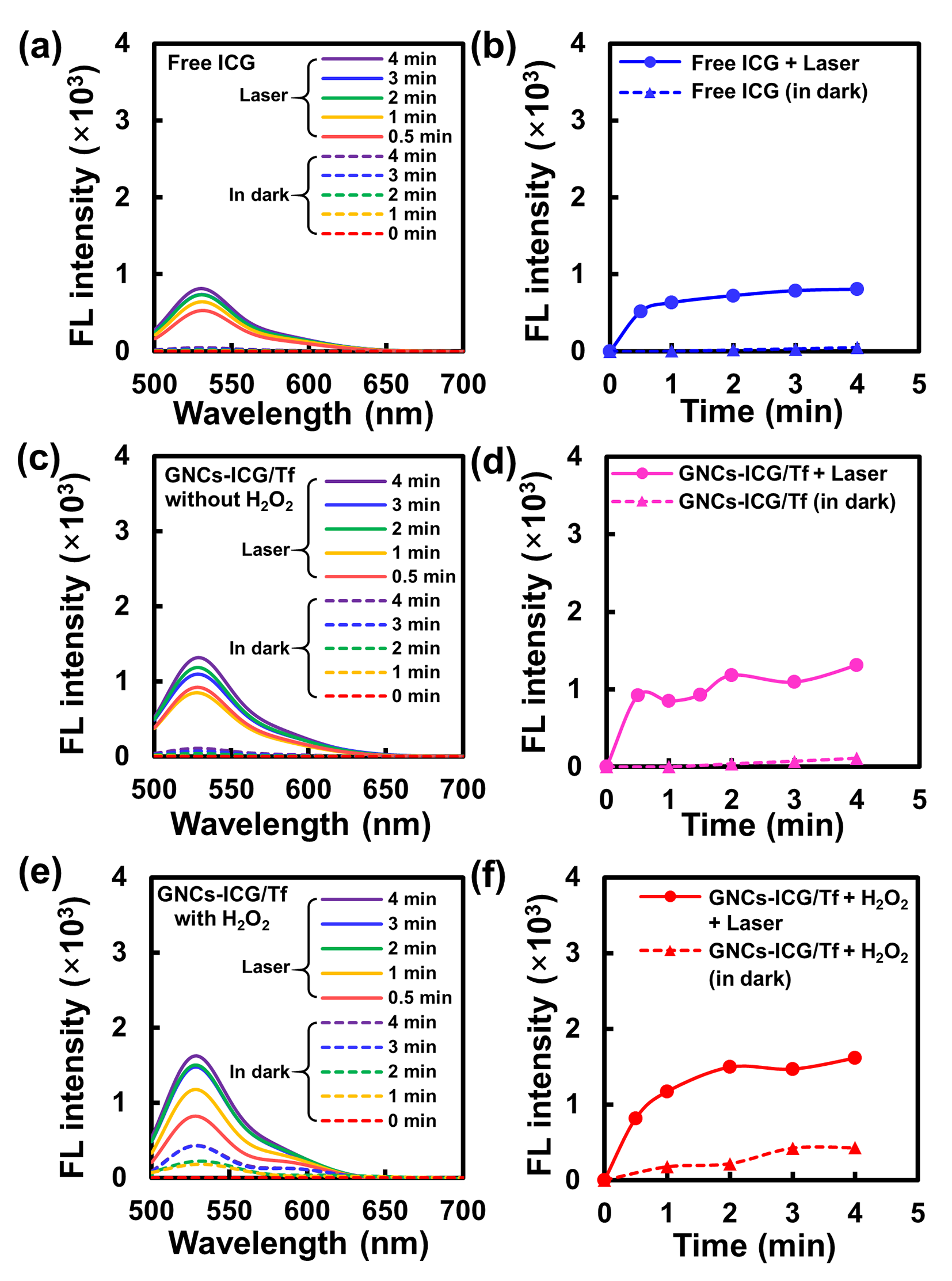


**Figure S19.** The ^1^O_2_ generation capability of free ICG (9.05 *μ*M) and GNCs-ICG/Tf (0.27 mg/mL). (a,b) Free ICG, (c,d) GNCs-ICG/Tf, and (e,f) GNCs-ICG/Tf + H_2_O_2_ (1.0%, v/v) with and without 808 nm laser (2 W/cm^2^).


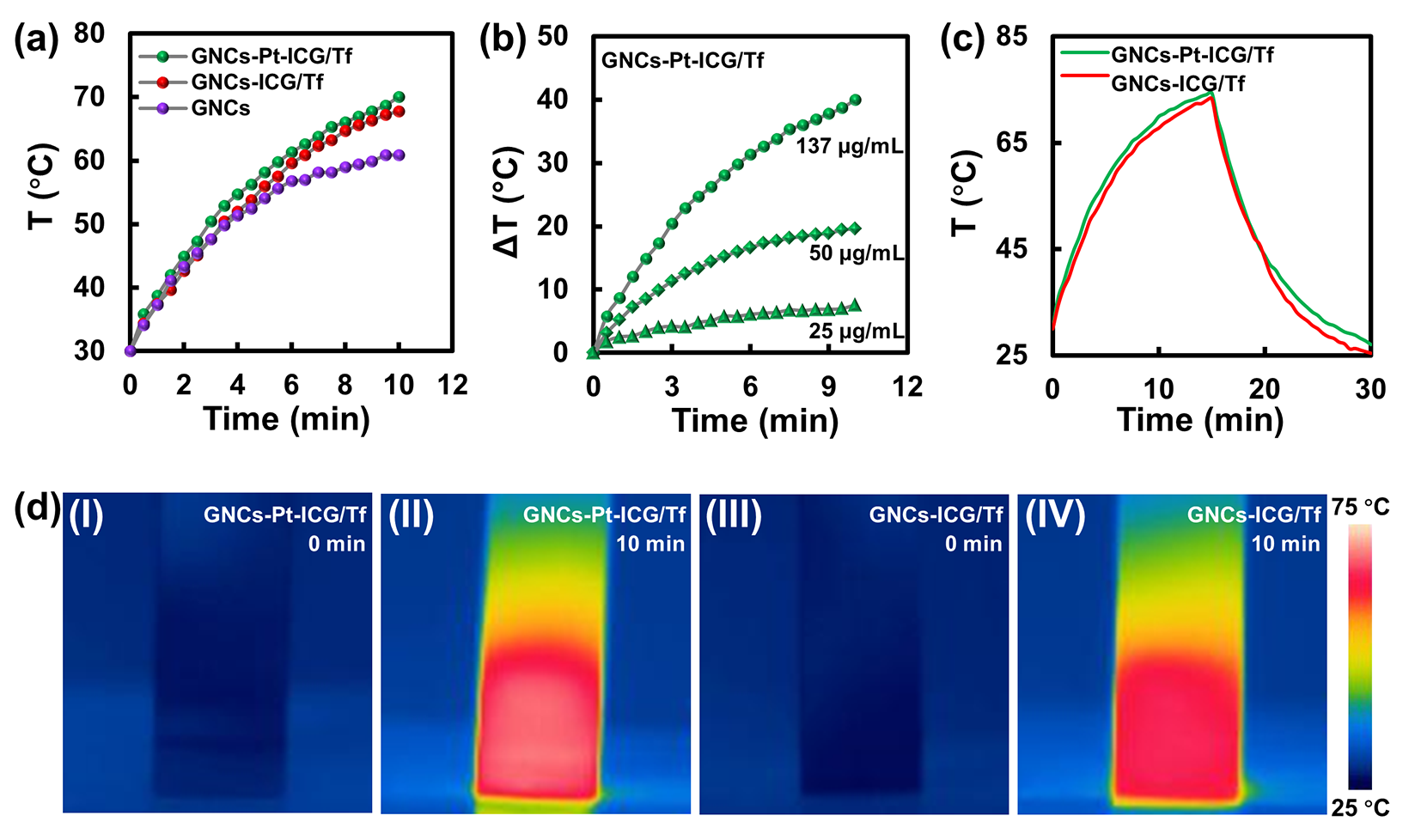


**Figure S20.** The photothermal effect of GNCs-Pt-ICG/Tf and GNCs-ICG/Tf. (a) Temperature evaluation of GNCs-Pt-ICG/Tf, GNCs-ICG/Tf, and GNCs with 808 nm laser irradiation for different times. (b) Temperature evaluation of GNCs-Pt-ICG/Tf at different concentrations with NIR laser irradiation for different times. (c) The photothermal effect of GNCs-Pt-ICG/Tf and GNCs-ICG/Tf dispersion in PBS under 808 nm laser, in which the irradiation lasted for 15 min and then turned off for another 15 min. The volume of solution is 1 mL. (d) The infrared thermal images of GNCs-Pt-ICG/Tf and GNCs-ICG/Tf under NIR irradiation at t = 0 and 10 min respectively. Laser: 808 nm, 2 W/cm^2^.

As shown in Figure S20a, under the same conditions, the temperature of GNCs is slightly lower than that of GNCs-Pt-ICG/Tf and GNCs-ICG/Tf, respectively. As shown in Figure 20b, the higher concentration of GNCs-Pt-ICG/Tf, the faster the heating rate was observed. As shown in Figure S20d, both the two infrared thermal images brightened rapidly upon NIR light irradiation, and the brightness of infrared thermal images of GNCs-Pt-ICG/Tf is slightly higher than that of GNCs-ICG/Tf, which was consistent with the temperature-time measurements (Figure 6e). The results indicated the efficient photothermal conversion capability of GNCs-Pt-ICG/Tf and the has the negligible influence of PtNPs deposition on the photothermal performance of GNCs.


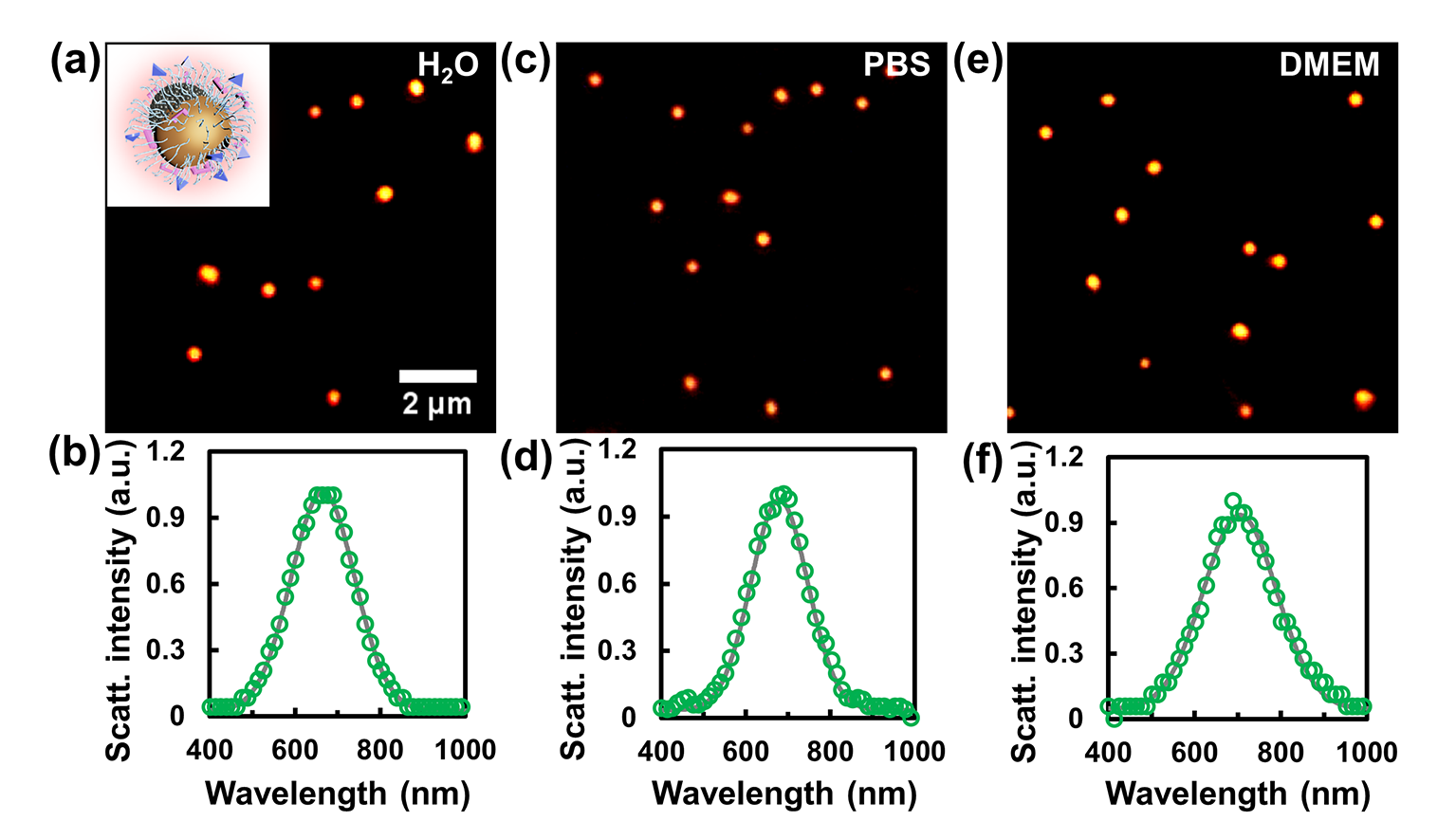


**Figure S21.** Stability of GNCs-Pt-ICG/Tf in different media. The dark-field optical microscopic images and single-particle scattering spectra of GNCs-Pt-ICG/Tf in (a,b) H_2_O, (c,d) PBS (10 mM, pH = 7.4), and (e,f) DMEM. Inset: schematic diagram of a GNC-Pt-ICG/Tf.


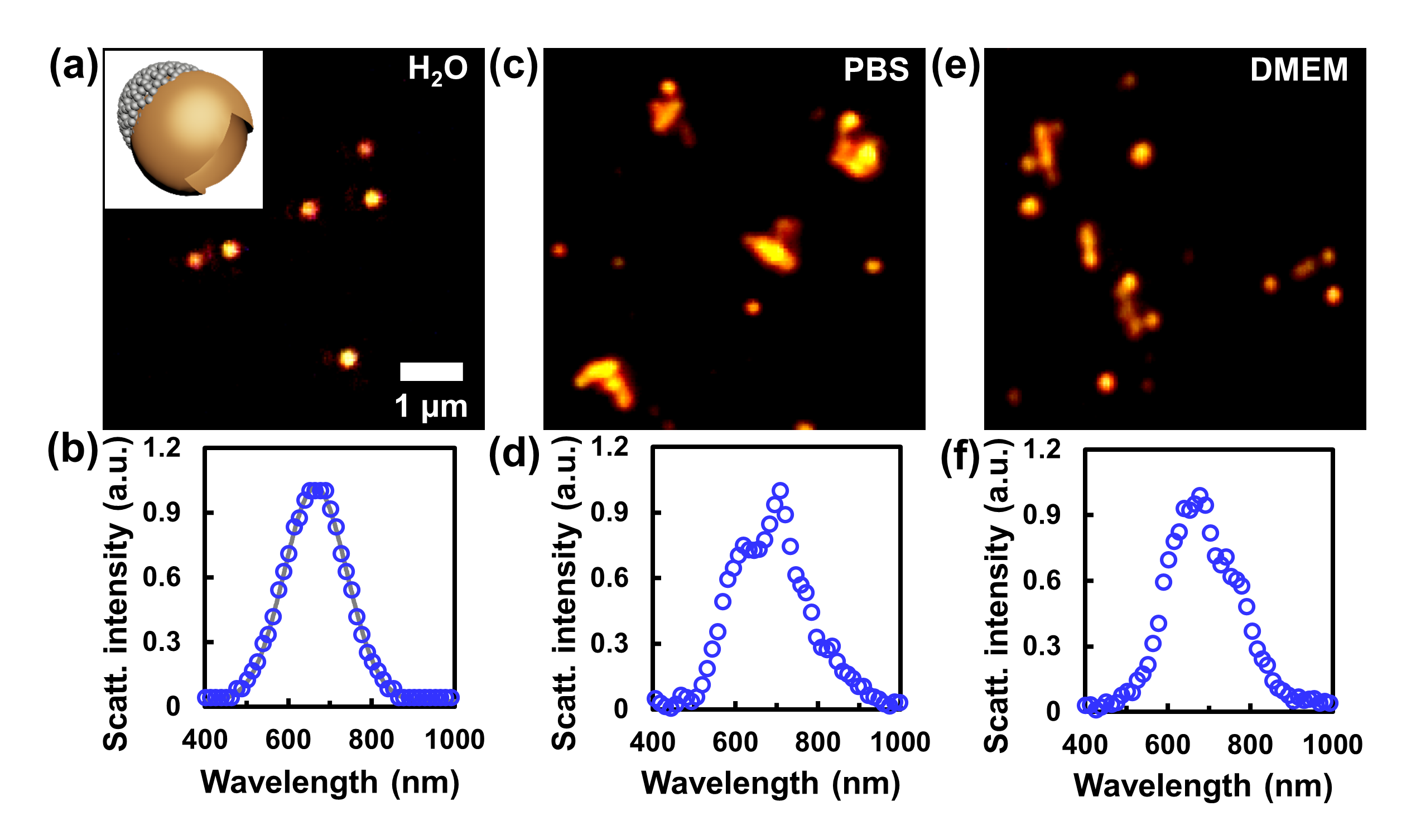


**Figure S22.** Stability of CTAB-stabilized GNCs-Pt in different media. The dark-field optical microscopic images and single-particle scattering spectra of CTAB-stabilized GNCs-Pt in (a,b) H_2_O, (c,d) PBS (10 mM, pH = 7.4), and (e,f) DMEM. Inset: schematic diagram of a CTAB-stabilized GNC-Pt.


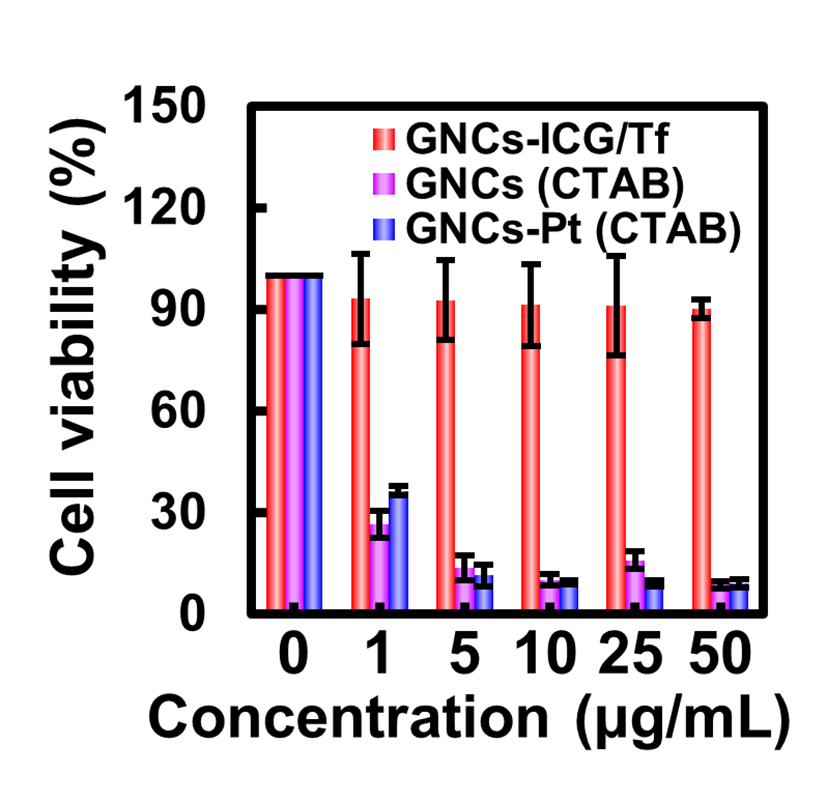


**Figure S23.** Cell viability of HepG2 cells after incubating with GNCs-ICG/Tf, CTAB-stabilized GNCs-Pt and GNCs at different concentrations (0, 1, 5, 10, 25, and 50 *μ*g/mL) in dark for 24 h respectively.


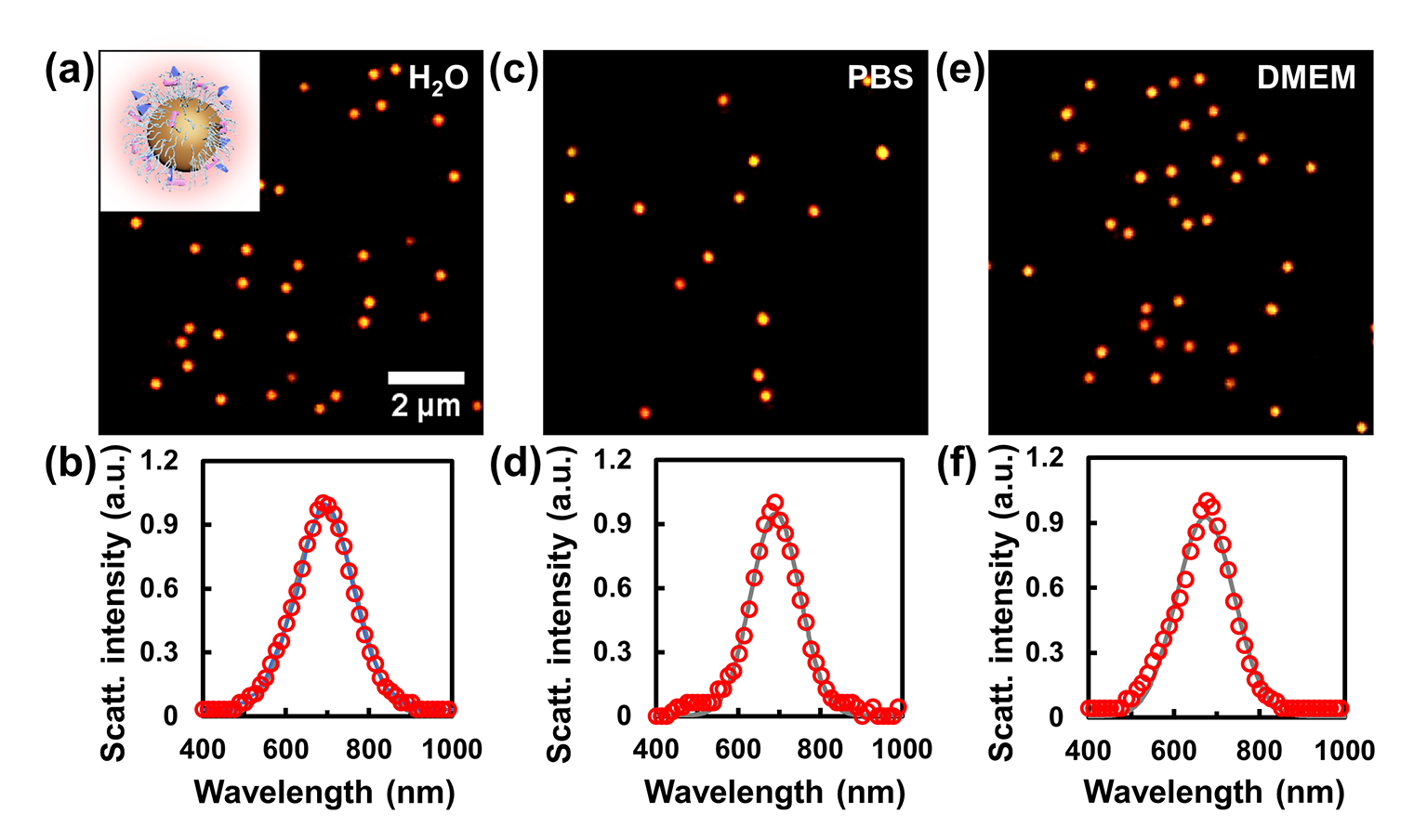


**Figure S24.** Stability of GNCs-ICG/Tf in different media. The dark-field optical microscopic images and single-particle scattering spectra of GNCs-ICG/Tf in (a,b) H_2_O, (c,d) PBS (10 mM, pH = 7.4), and (e,f) DMEM. Inset: schematic diagram of a GNC-ICG/Tf.


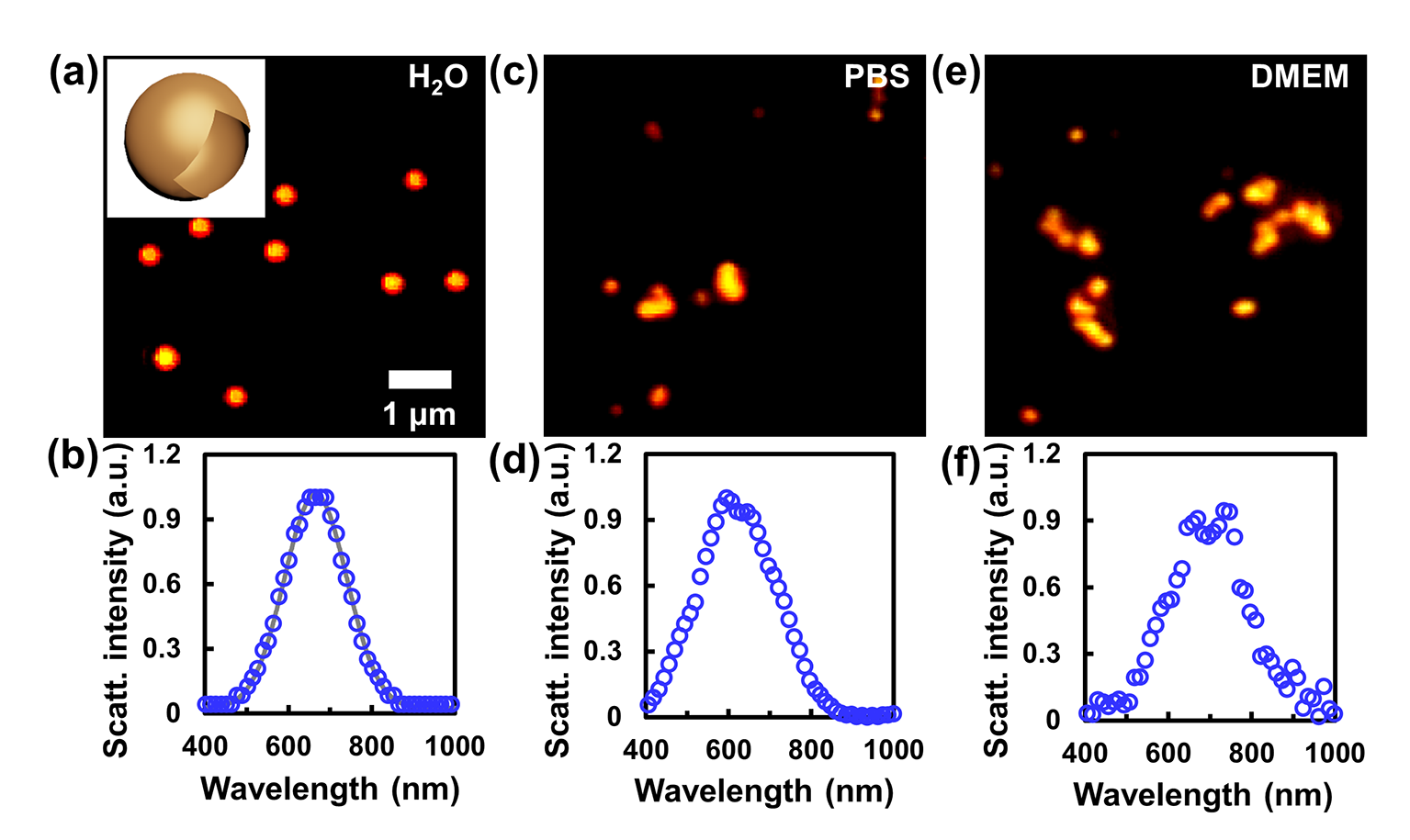


**Figure S25.** Stability of CTAB-stabilized GNCs in different media. The dark-field optical microscopic images and single-particle scattering spectra of CTAB-stabilized GNCs in (a,b) H_2_O, (c,d) PBS (10 mM, pH = 7.4), and (e,f) DMEM. Inset: schematic diagram of a CTAB-stabilized GNC.


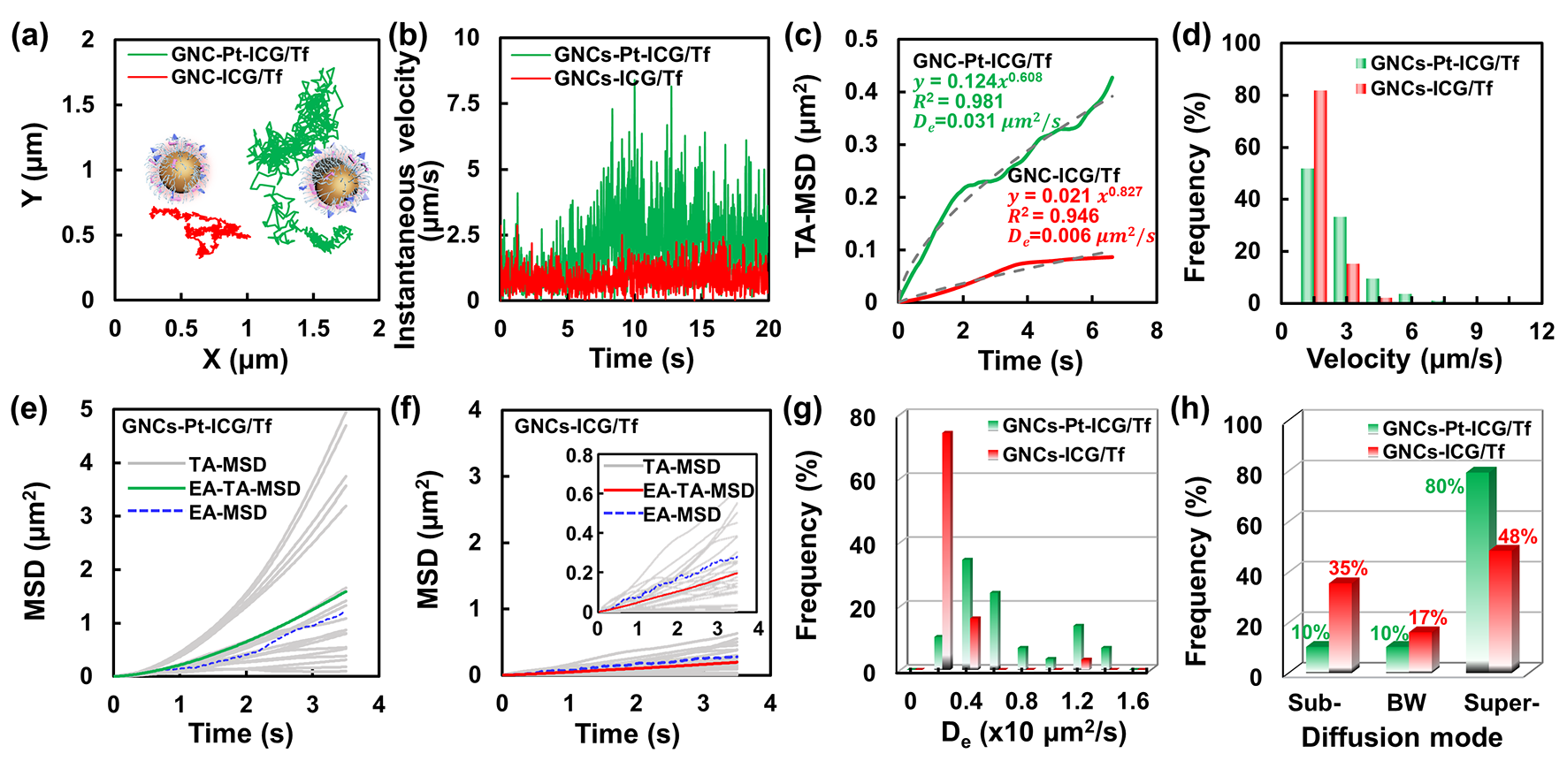


**Figure S26.** The motion behaviors of GNCs-Pt-ICG/Tf and GNCs-ICG/Tf on HepG2 cell membrane. (a) Trajectories (in 20 s period), (b) instantaneous velocity ($v$), and (c) TA-MSD of GNCs-Pt-ICG/Tf and GNCs-ICG/Tf on HepG2 cell membrane. Inset: schematic diagrams of individual GNC-Pt-ICG/Tf and GNC-ICG/Tf. (d) Distributions of instantaneous velocity of GNCs-Pt-ICG/Tf and GNCs-ICG/Tf. (e,f) MSD of GNCs-Pt-ICG/Tf and GNCs-ICG/Tf, respectively. The solid lines (gray) represent the TA-MSD curves of 30 individual trajectories, respectively. The thick lines represent the EA-TA-MSD (green for GNCs-Pt-ICG/Tf, and red for GNCs-ICG/Tf). The dashed curves (blue) denote the EA-MSD calculated from all individual trajectories. Distributions of (g) $D_{e}$ and (h) diffusion modes (including sub-diffusion, Brownian motion (BW), and super-diffusion) of GNCs-Pt-ICG/Tf and GNCs-ICG/Tf.

For the clarity of presentation, we only show representative trajectories in the Figure S26a. The statistical results were also displayed in Figures S26c-h. The instantaneous velocity, and $D_{e}$ of GNCs-Pt-ICG/Tf are larger than those of GNCs-ICG/Tf. These results indicate the movability of GNCs-Pt-ICG/Tf is enhanced, which can facilitate the wide search and efficient recognition for tumor targets.

As shown in Figures S26e and f, no matter GNCs-Pt-ICG/Tf nor GNCs-ICG/Tf, EA-TA-MSD is inconsistent with TA-MSD and EA-MSD. The deviation between EA-TA-MSD and EA-MSD of GNCs-Pt-ICG/Tf is more significant than that of GNCs-ICG/Tf. These results indicate that the more significant heterogeneity not only exists among individual GNCs-Pt-ICG/Tf, but also exists at different time for the same GNCs-Pt-ICG/Tf.


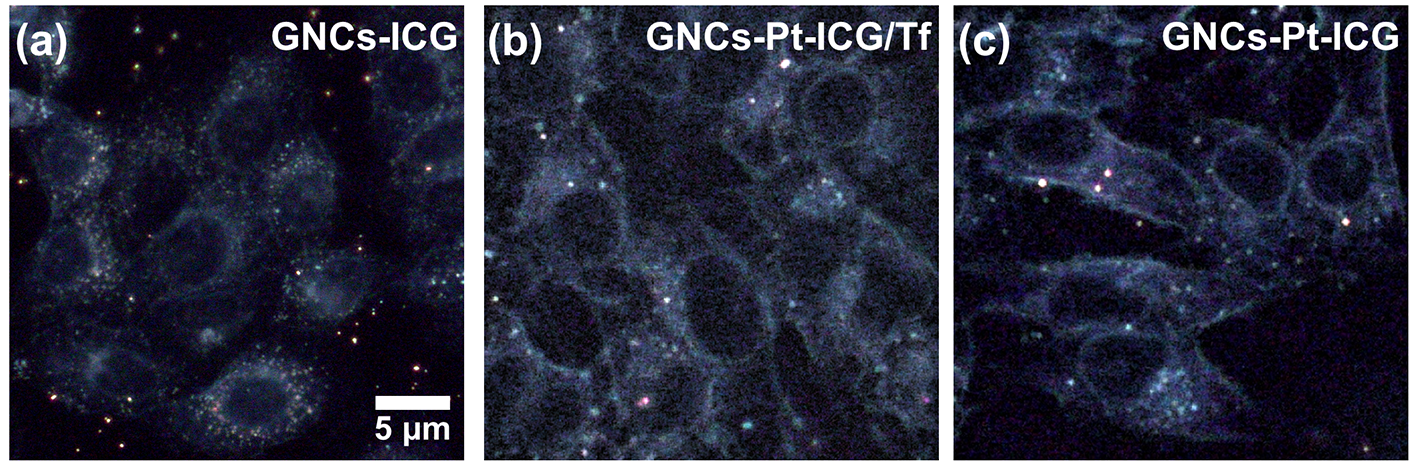


**Figure S27.** Uptake efficiency of different nanomaterials for living cells. (a) GNCs-ICG for HepG2 cells. (b) GNCs-Pt-ICG/Tf, and (c) GNCs-Pt-ICG for NCTC1469 cells (a mouse fibroblasts cell line, a normal cell with negligible surface expression of TfR).

The number of GNCs-Pt-ICG/Tf in the NCTC1469 cells (a mouse fibroblasts cell line) is much less than in the HepG2 cells, indicating the specific recognition ability of GNCs-Pt-ICG/Tf toward cancerous cells. Due to the promotion of Tf in specific recognition for HepG2 cells, we further explored whether Tf facilitates the binding to non-cancer cells. The uptake of GNCs-Pt-ICG/Tf and GNCs-Pt-ICG by NCTC1469 cells was far lower than that of corresponding HepG2 cells respectively. There was no significant difference in the NCTC1469 cellular uptake for the two groups. These results further supported the great selectivity for cancer cells as well as pretty low side effects on non-cancer cells of GNCs-Pt-ICG/Tf.

**Supplementary tables**

**Table S1**. $K_{m}$ and $V_{max}$ of GNCs-Pt towards TMB and H_2_O_2_.


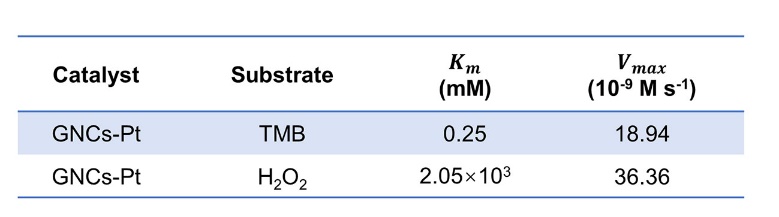


**Table S2.** Fitting the curves of EA-TA-MSD versus the time interval (∆t) of GNCs-Pt with different H_2_O_2_ concentrations (0, 1, 2, 3, 5, and 10%). Equation $TE-\mathrm{MSD}\left( \Delta t \right)=2dD_{e}{\Delta t}^{\alpha}, (d=2$) is employed to estimate the effective diffusion coefficient ($D_{e}$) and anomalous exponent ($\alpha$).


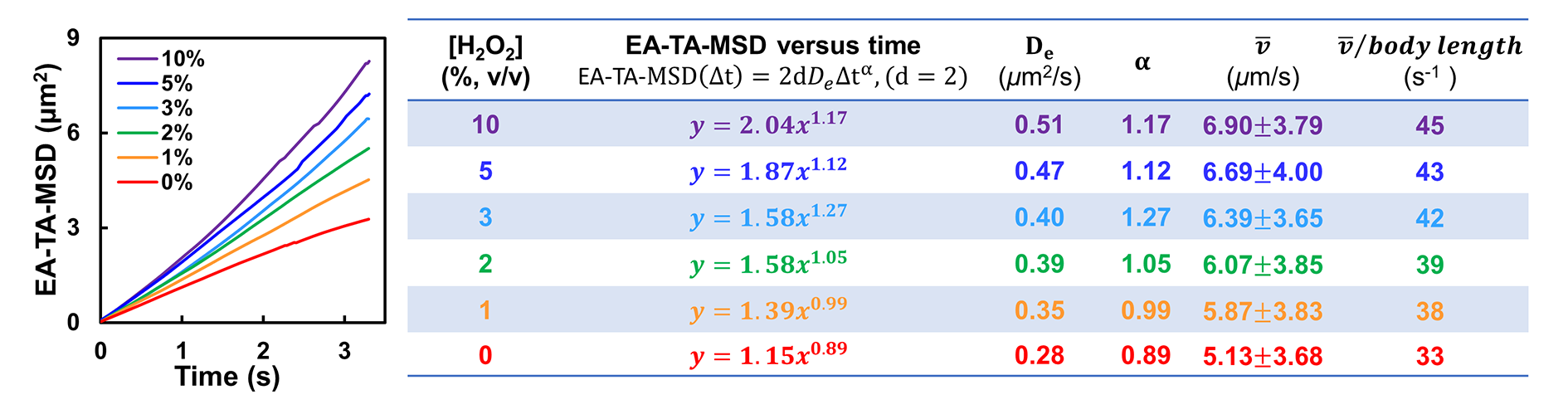


**Table S3.** FT-IR analysis of GNCs-Pt-ICG/Tf (mPEG-SH) and GNCs-ICG/Tf (mPEG-SH).


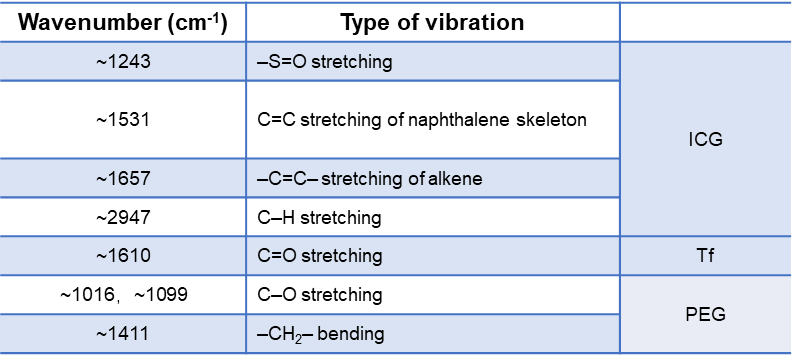


**Supplementary references**

[1] Jiang, R.; Qin, F.; Liu, Y., et al. "Colloidal gold nanocups with orientation-dependent plasmonic properties," *Adv. Mater.*, vol. *28,* no. (30), pp. 6322-6331, 2016.

[2] Yang, H.; Xu, B.; Li, S., et al. "A photoresponsive nanozyme for synergistic catalytic therapy and dual phototherapy," *Small*, vol. *17,* no. (10), pp. e2007090, 2021.

[3] Wei, F.; Cui, X.; Wang, Z., et al. "Recoverable peroxidase-like Fe_3_O_4_@MoS_2_-Ag nanozyme with enhanced antibacterial ability," *Chem. Eng. J.*, vol. *408,* no., pp. 127240, 2021.

[4] Wan, M.; Chen, H.; Wang, Q., et al. "Bio-inspired nitric-oxide-driven nanomotor," *Nat. Commun.*, vol. *10,* no. (1), pp. 966, 2019.

[5] Wang, W.; Chiang, T.-Y.; Velegol, D.; Mallouk, T. E. "Understanding the efficiency of autonomous nano- and microscale motors," *J. Am. Chem. Soc.*, vol. *135,* no. (28), pp. 10557-10565, 2013.

[6] Lee, T.-C.; Alarcón-Correa, M.; Miksch, C., et al. "Self-propelling nanomotors in the presence of strong brownian forces," *Nano Lett.*, vol. *14,* no. (5), pp. 2407-2412, 2014.

[7] Mou, F.; Chen, C.; Zhong, Q., et al. "Autonomous motion and temperature-controlled drug delivery of Mg/Pt-poly(n-isopropylacrylamide) Janus micromotors driven by simulated body fluid and blood plasma," *ACS Appl. Mater. Interfaces*, vol. *6,* no. (12), pp. 9897-9903, 2014.

[8] Ye, Z.; Wang, X.; Xiao, L. "Single-particle tracking with scattering-based optical microscopy," *Anal. Chem.*, vol. *91,* no. (24), pp. 15327-15334, 2019.

[9] Manzo, C.; Garcia-Parajo, M. F. "A review of progress in single particle tracking: From methods to biophysical insights," *Rep. Prog. Phys.*, vol. *78,* no. (12), pp. 124601, 2015.

[10] Choi, C. H. J.; Alabi, C. A.; Webster, P.; Davis, M. E. "Mechanism of active targeting in solid tumors with transferrin-containing gold nanoparticles," *Proc. Natl. Acad. Sci. U. S. A.*, vol. *107,* no. (3), pp. 1235-1240, 2010.

[11] Ye, Z.; Wei, L.; Zeng, X., et al. "Background-free imaging of a viral capsid proteins coated anisotropic nanoparticle on a living cell membrane with dark-field optical microscopy," *Anal. Chem.*, vol. *90,* no. (2), pp. 1177-1185, 2018.

[12] Higbee-Dempsey, E.; Amirshaghaghi, A.; Case, M. J., et al. "Indocyanine green-coated gold nanoclusters for photoacoustic imaging and photothermal therapy," *Adv. Therap.*, vol. *2,* no. (9), pp. 1900088, 2019.

[13] Li, W.; Zhang, H.; Guo, X., et al. "Gold nanospheres-stabilized indocyanine green as a synchronous photodynamic-photothermal therapy platform that inhibits tumor growth and metastasis," *ACS Appl. Mater. Interfaces*, vol. *9,* no. (4), pp. 3354-3367, 2017.

[14] Zhou, M.; Huang, H.; Wang, D., et al. "Light-triggered pegylation/depegylation of the nanocarriers for enhanced tumor penetration," *Nano Lett.*, vol. *19,* no. (6), pp. 3671-3675, 2019.
